# Neutrophils drive accelerated tumor progression in the collagen-dense mammary tumor microenvironment

**DOI:** 10.1186/s13058-016-0703-7

**Published:** 2016-05-11

**Authors:** María G. García-Mendoza, David R. Inman, Suzanne M. Ponik, Justin J. Jeffery, Dagna S. Sheerar, Rachel R. Van Doorn, Patricia J. Keely

**Affiliations:** Department of Cell and Regenerative Biology, University of Wisconsin – Madison, Madison, WI USA; UW Carbone Cancer Center, University of Wisconsin – Madison, Madison, WI USA; Wisconsin Institutes of Medical Research, 1111 Highland Ave., Madison, WI 53705 USA; Present Address: Department of Epigenetics and Molecular Carcinogenesis, The University of Texas MD Anderson Cancer Center, Houston, Texas USA

**Keywords:** Extracellular matrix, Tumor microenvironment, Collagen, Tumor associated neutrophils, Breast cancer, MMTV-PyVT, Stroma, Cytokines

## Abstract

**Background:**

High mammographic density has been correlated with a 4-fold to 6-fold increased risk of developing breast cancer, and is associated with increased stromal deposition of extracellular matrix proteins, including collagen I. The molecular and cellular mechanisms responsible for high breast tissue density are not completely understood.

**Methods:**

We previously described accelerated tumor formation and metastases in a transgenic mouse model of collagen-dense mammary tumors (type I collagen-α1 (Col1α1)^tm1Jae^ and mouse mammary tumor virus - polyoma virus middle T antigen (MMTV-PyVT)) compared to wild-type mice. Using ELISA cytokine arrays and multi-color flow cytometry analysis, we studied cytokine signals and the non-malignant, immune cells in the collagen-dense tumor microenvironment that may promote accelerated tumor progression and metastasis.

**Results:**

Collagen-dense tumors did not show any alteration in immune cell populations at late stages. The cytokine signals in the mammary tumor microenvironment were clearly different between wild-type and collagen-dense tumors. Cytokines associated with neutrophil signaling, such as granulocyte monocyte-colony stimulated factor (GM-CSF), were increased in collagen-dense tumors. Depleting neutrophils with anti-Ly6G (1A8) significantly reduced the number of tumors, and blocked metastasis in over 80 % of mice with collagen-dense tumors, but did not impact tumor growth or metastasis in wild-type mice.

**Conclusion:**

Our study suggests that tumor progression in a collagen-dense microenvironment is mechanistically different, with pro-tumor neutrophils, compared to a non-dense microenvironment.

**Electronic supplementary material:**

The online version of this article (doi:10.1186/s13058-016-0703-7) contains supplementary material, which is available to authorized users.

## Background

Although much breast cancer research focuses on the genetic abnormalities seen in cancer cells compared to normal breast epithelial cells, emerging data reveal that components of the extracellular matrix (ECM) and cells in the microenvironment are key regulators of tumor progression and metastasis. Women with 75 % or greater mammographically dense breast tissue are four to six times more likely to develop breast carcinoma than women with less than 10 % mammographically dense tissue [1, 2]. As the molecular and cellular mechanisms responsible for high breast tissue density are not completely understood, many studies rely on in vivo and in vitro models of density to discover new preventative, prognostic, and therapeutic targets that will reduce breast cancer risk in patients with high breast tissue density. Much of the in vitro work has focused on the response of mammary epithelial cells to collagen-dense matrices in the regulation of proliferation and invasiveness [3, 4].

It has been shown that mammographic tissue density corresponds to alterations in stromal composition [5–7]. High mammographic tissue density is largely attributed to increased levels of the ECM protein, collagen [8–11]. To better understand the role of increased collagen density, we utilize a mouse model in which the *Collagen 1α1* gene is mutated to make the molecule resistant to collagenase, resulting in decreased collagen turnover and a net increase in stromal collagen (Col1α1^tm1Jae^) [12]. These animals are crossed to the mouse mammary tumor virus-polyoma middle T antigen (MMTV-PyVT) model, which is commonly used because it is comparable with human breast disease, it progresses from premalignant to malignant tumor and to lung metastasis. Not only is the morphology similar to that in human disease, but also the biomarkers expressed in PyVT tumors are consistent with those associated with poor outcome in humans [13, 14]. PyVT tumors arising in the collagen-dense (COL) Col1α1 background have a three-fold increase in tumor formation and lung metastasis compared to tumors arising in wild-type (WT) mice. The exact mechanism by which increased collagen deposition leads to increased metastasis is not entirely clear. However, we previously noted an increase in the stromal cell populations surrounding tumors within collagen-dense environments, suggesting activation of the stromal compartment [12].

The breast tumor microenvironment is composed of ECM proteins and both malignant and non-malignant cells. Of the non-malignant, CD45+ immune cells, both innate and adaptive cells are present in the tumor microenvironment. T cells (CD8+ cytotoxic cells, CD4+ helper T cells, γδ T cells) and natural killer (NK) cells play vital anti-tumor roles before tumor cells are able to evade immune surveillance [15, 16]. Myeloid cells, on the other hand, have been shown to often have pro-tumor functions in breast cancer. Tumor cells can educate and influence macrophages via specific cytokine signaling crosstalk [17]. Tumor-associated macrophages (TAMs) can enhance tumor cell migration and invasion, stimulate angiogenesis, remodel the ECM, and aid breast cancer metastasis [18–20]. Tissue studies from prophylactic mastectomies show that highly mammographically dense tissue is characterized by decreased alternatively activated (M2) macrophages in the stroma and CD45+ immune cells in the epithelium [10].

Emerging evidence also suggests neutrophils may be active players in cancer progression. Similar to macrophages, but much less understood in breast cancer, neutrophils are thought to promote tumor growth by reducing proinflammatory factors, remodeling the ECM with proteases that also aid in angiogenesis and increasing metastasis [21–23]. Tumor-associated neutrophils (TANs), in addition to TAMs, can reduce cytotoxic T cell activity that would kill tumor cells, leading to tumor growth [24]. TANs contribute to angiogenesis through matrix metalloproteinase 9 (MMP-9) in human fibrosarcoma and prostate cancer cells [25]. Neutrophil involvement in metastasis in different breast cancer models has been uncertain due to conflicting results [26]. In the PyVT model, depleting neutrophils increases the number of metastases per lung [27]. In contrast, depletion of neutrophils in the orthotopic 4T1 mouse mammary carcinoma decreases the number of lung metastases [28].

Here we investigate the non-malignant, immune cells present in the collagen-dense tumor microenvironment that may promote tumor progression and metastasis. In this study, we report that a pro-tumor immune cell and cytokine profile characterize the collagen-dense mouse mammary tumor microenvironment. We find an inherent difference in certain cytokine levels in WT versus collagen-dense tumors. These signals support the recruitment and activation of neutrophils in the collagen-dense tumor microenvironment. Our results suggest that a collagen-dense tumor microenvironment can tip the balance between a tumor promoting and tumor suppressing phenotype of neutrophils. Depleting neutrophils significantly slowed the formation of new tumors, and reduced tumor burden and lung metastasis only in tumors arising in the collagen-dense tumor microenvironment, but not in WT MMTV-PyVT mice. These findings suggest that tumor progression in a collagen-dense microenvironment, compared to non-dense microenvironments, occurs through a distinct subpopulation of immune cell effectors.

## Methods

### Mice

Mice were bred and maintained at the University of Madison – Wisconsin under the approval of the University of Wisconsin Animal Care and Use Committee (approved animal protocol number: M01668). Transgenic male mice expressing the polyoma virus middle T antigen under the direction of the mammary mouse tumor virus promoter (MMTV-PyVT) in the Friend virus B type (FVB) background (originated from The Jackson Laboratory, Bar Harbor, ME, USA) were used as a model of breast cancer that develops spontaneous tumors and metastasizes to the lung [14]. These mice were crossed to female mice heterozygote for the *Col1α1* mutation in the C57BL/6/129 background (originated from The Jackson Laboratory). The *Co1α1* mutation renders the alpha 1 chain of collagen I uncleavable by collagenase and increases collagen in the tissue due to decreased remodeling [29]. The resulting mice were either positive for the MMTV-PyVT (tumor mice) or negative (normal mice), and they were either WT for the *Col1α1* mutation (WT) or heterozygote (COL). Genotyping by polymerase chain reaction (PCR) was performed on DNA extracted from tail biopsies.

### Tissue collection

Female mice were examined for palpable tumors starting at 8 weeks. At 15 weeks of age, matched sibling pairs of WT and COL mice with tumors were anesthetized with 4 % isofluorane. Blood was collected via retro-orbital procedure into an ethylenediaminetetraacetic acid (EDTA) (1.3 mg/ml of blood) collection tube (Sarstedt, Numbrecht, Germany). Mice were then perfused intracardially with 0.9 % sodium chloride (Abbott Laboratories, Chicago, IL, USA) supplemented with 1 unit of heparin (Hospira, Lake Forest, IL, USA). Mammary gland tumors and spleens were harvested and placed in high glucose DMEM (Gibco, Grand Island, NY, USA) supplemented with 10 % fetal bovine serum (FBS, Gemini Bio Products, Baltimore, MD, USA) and 1× antibiotic antimycotic solution (Corning, Corning, NY, USA) for ELISA cytokine array and flow cytometry experiments. Tumors from the fourth or fifth (right or left) mammary glands and the lungs were harvested and fixed in 10 % buffered formalin (Thermo Fisher Scientific, Kalamazoo, MI, USA). Spleens were weighed and cut in half: one half was placed in DMEM with 10 % FBS and 1× antibiotic antimycotic for flow cytometry experiments, and the other half was fixed in 10 % buffered formalin. Tumors were weighed and approximately 150 mg of tumor was used per mice per flow cytometry experiment.

### ELISA plate array

To prepare the cell lysate, 100 mg of fresh tumor from WT and COL tumor mice was placed in 1 ml of cell lysis buffer (Signosis, Inc., Sunnydale, CA, USA). Tumors were homogenized on ice in a PowerGen 125 homogenizer (Fisherbrand, Pittsburgh, PA, USA) for one minute. Lysates were sonicated at 20 % power for 10 seconds three times using a Sonic Dismembrator Model 500 (Fisher Scientific) with a Branson tip Model 102 converter. The samples were centrifuged at 10,000 RPM for 5 minutes. The supernatant was then aliquoted and frozen at −80 °C until ready to use.

Mouse Cytokine ELISA Plate Array (Chemiluminescence) (Signosis, Inc.) is a 96-well plate divided into four sections; each section has three columns for one sample. Each section also has 23 specific cytokine capture antibodies coated onto each well and one blank well. Samples were thawed and diluted to 10 μg per 100 μl per well. Briefly, each well was incubated with 100 μl of sample for 2 hours, followed by biotin-labeled antibody mixture for 1 hour with gentle shaking, then with strepavidin-HRP conjugate for 30 minutes with gentle shaking, then finally with substrate solution for 2 minutes. The plate was read on a Fluoroskan Ascent™ FL microplate fluorometer using the Ascent™ Software (Thermo Scientific, Waltham, MA, USA).

### Single cell isolation

Single cells were obtained by cutting tumors and spleens into small pieces and digesting tumors with 0.028 W/ml of Liberase TM Research Grade (Roche, Mannheim, Germany), 20 μg/ml of DNase I, and 1× antibiotic antimycotic in DMEM with 10 % FBS for 1 hour at 37 °C with 250 RPM agitation. Digestion was facilitated by pipetting up and down every 15 minutes. The cell suspension was filtered through a 70-μm and 40-μm nylon cell strainer (BD Biosciences, Franklin Lakes, NJ, USA). Red blood cells were lysed from tumor and spleen single cell suspensions with lysis buffer (Sigma, St. Louis, MO, USA), and cells were resuspended in wash buffer (3 % FBS in PBS, Gibco, Grand Island, NY, USA).

### Flow cytometry

Cells were resuspended at 10^6^ cells per 100 μl of wash buffer and blocked with Fc block (anti CD16/CD32, BD Biosciences) for 5 minutes at 4 °C. For the myeloid cell flow panel, 10^6^ cells were incubated with Alexa Fluor® 488-F4/80 (clone BM8, Invitrogen, Carlsbad, CA, USA), phycoerythrin (PE)-CCR2 (clone 475301, R&D Systems, Minneapolis, MN, USA), PE-Cy™5-CD45 (clone 30-F11), Brilliant Violet 711™-CD3ε (clone 145-2C11), allophycocyanin (APC)-Ly6C (clone, AL-21) from BD Biosciences, Brilliant Violet 421™-Ly6G (clone 1A8, BioLegend, San Diego, CA, USA), and Alexa Fluor® 700-CD11b (clone M1/70, eBiosciences, San Diego, CA, USA) for 30 minutes at 4 °C in the dark. Other antibodies used include: fluorescein isothiocyanate (FITC)-CD335 (NKp46) (clone 29A1.4) and APC-CD49b (clone DX5) from BD Biosciences, and PE-F4/80 (clone CI:A3-1, AbD Serotec, Kidlington, UK). For the lymphocyte flow cytometry panel, 10^6^ cells were incubated with PE-CD4 (clone GK1.5), PE-Cy™5-CD45 (clone 30-F11), PE-Cy™7-CD25 (clone PC61), Brilliant Violet 711™-CD3ε (clone 145-2C11), APC-CD19 (clone, 1D3) from BD Biosciences, Brilliant Violet 421™-CD8a (clone 53–6.7, BioLegend), Alexa Fluor® 700-CD11b (clone M1/70, eBiosciences, San Diego, CA, USA) for 30 minutes at 4 °C. After washing, cells were stained with 1 μl of Fixable Viability Dye eFluor® 780 (eBiosciences) for 30 minutes at 4 °C in the dark. Cell samples from the lymphocyte panel were stained with the Foxp3/transcription factor staining buffer set from eBiosciences. Briefly, cells were incubated in 1 ml of the fixation/permeabilization working solution for 30 minutes at 4 °C; cells were washed and left in 1× permeabilization buffer overnight at 4 °C in the dark. Cells were resuspended in 100 μl of 1× permeabilization buffer and stained with Alexa Fluor® 488-Foxp3 (clone FJK-16 s, eBiosciences) for 30 minutes at room temperature in the dark. After the final wash, all cells were resuspended in 500 μl of wash buffer for analysis.

Samples were run on the LSRII and LSR Fortessa (BD Biosciences) benchtop flow cytometers at the University of Wisconsin Carbone Cancer Center (UWCCC) Flow Cytometry Facility. Each experiment was standardized using Sphero™Rainbow Fluorescent Particles (Mid-Range) (Spherotech Inc., Lake Forest, IL, USA). LSRII and LSR Fortessa sensitivities were optimized during the initial run of the experiment. Rainbow beads were collected at those optimized settings and median fluorescent intensity (MFI) target values were recorded in each channel. For every run, rainbow beads were acquired first. LSRII and LSR Fortessa voltages were adjusted until the bead peaks hit the MFI target value (±10 %). The instrument was compensated using UltraComp eBeads (eBiosciences). For each tumor sample, 25,000 live, single cell events were collected, and for each spleen sample, 50,000 live, single cell events were collected. Data were analyzed using FlowJo Single Cell Analysis Software (TreeStar, Ashland, OR, USA), and gating was established using fluorescent minus one (FMO) controls.

### Immunohistochemical analysis

Tissues (mammary tumor, mammary gland, spleen, and lungs) were fixed in 10 % buffered formalin (Thermo Fisher Scientific) for 48 hours. These were then placed in 70 % ethanol and taken to the UWCCC Experimental Pathology Laboratory (EPL) for processing. Tissues were embedded in paraffin and sectioned, and one section was stained with hematoxylin and eosin (H&E) at the EPL. COL and WT tumor sections were deparaffinized by heating at 60 °C for 25 minutes. They were then placed in xylenes and rehydrated in gradual dilutions of ethanol. Antigen retrieval was done in citrate buffer pH 6.0 in a boiling water bath for 15 minutes. Endogenous horseradish peroxidase (HRP) activity was blocked by incubating sections in 0.3 % H_2_O_2_ in Tris-buffered saline (TBS) for 20 minutes at room temperature. Sections were blocked with 5 % normal serum and 1 % BSA in TBS (blocking solution) for 1 hour, and with Avidin/Biotin Block (ABC, Vector Laboratories, Burlingame, CA, USA) for 15 minutes each. Sections were incubated with primary rat-anti-mouse Ly6G (1:1000, clone 1A8, Biolegend) for 2 hours, and with affinity purified, mouse absorbed, biotinylated anti-rat secondary IgG (Vector Laboratories) for 45 minutes. All incubations were done at room temperature. Sections were incubated with Vectastain Universal Ready-to-Use ABC reagent (Vector Laboratories) for 30 minutes, stained with 3,3’ diaminobenzidine (DAB) and counterstained with hematoxylin (Leica, Nussloch, Germany). Sections were dehydrated in gradual dilutions of ethanol and xylenes before mounting in Richard Allen Scientific mounting medium (Thermo Fisher).

Slides were imaged at the University of Wisconsin – Madison Translational Research Initiatives in Pathology Laboratory using the Nuance™ multispectral imaging system (PerkinElmer, Waltham, MA, USA). Eight fields per tumor slide were captured with the 40x objective using the Nuance™ analysis software. Ly6G-positive cells were counted manually for each image and averaged per tumor.

### Neutrophil depletion

Mice in the treated group received intra-peritoneal injections of 5.5 μg/g of *InVivo*Mab anti mouse Ly6G (clone 1A8, BioXCell, West Lebanon, NH, USA) and mice in the control group received 5.5 μg/g of *InVivo*Mab rat IgG2a (clone 2A3, BioXCell) to deplete neutrophils in the COL and WT tumor mice. Injections were administered every 3 days for 24 days. Mice were 9 weeks of age at the beginning and 12 weeks of age at the conclusion of the experiment. Systemic neutrophil depletion was evaluated periodically by collecting blood samples from the saphenous vein. Samples were analyzed in a Hemavet 950FS (Drew Scientific, Waterbury, CT, USA) whole blood counter.

Hybrid positron emission tomography (PET) and computed tomography (CT) mouse imaging before and at the end of treatment was conducted at the UWCCC Small Animal Imaging Facility. All mice were fasted for 12 hours prior to intravenous injection of approximately 8 MBq of 2′-deoxy-2′-[^18^F]fluoro-d-glucose (^18^FDG) (IBA-Molecular, Sterling, VA, USA) 1 hour before imaging. Mice were anesthetized with inhalation gas (2 % isoflurane gas mixed with 1 L/min of pure oxygen) and kept under a heat lamp during injection until imaging. Mice were imaged on the Siemens Inveon Hybrid micro-PET/CT (Siemens Medical Solutions, Knoxville, TN, USA) in the prone position. A 10-minute PET scan was acquired and data were histogrammed into one static frame and subsequently reconstructed using ordered-subset expectation maximization (OSEM) of three dimensions followed by the maximum *a posteriori* algorithm (matrix size = (128,128,159), pixel size = (0.776, 0.776, 0.796) mm, iterations = 18, subsets = 16, and beta smoothing factor = 0.004). Data were not corrected for attenuation or scatter.

Data were analyzed using the Siemens Inveon™ Research Workplace (Siemens Medical Solutions). Source CT 3D images were co-registered with target PET 3D images. Date, time, and dose (Mbq) were entered for each data point (mouse). PET and CT scales were set at specific minimum and maximum percent injection dose per gram (%ID/g) and applied to every data point. Regions of interest (ROIs) were drawn at any mammary gland that had an FDG uptake threshold value between 7.5 and 27%ID/g based on background region of interest (ROI) reading (muscle) and image contrast. ROI data collected included volume (mm^3^), mean %ID/g, minimum %ID/g, and maximum %ID/g. Maximum intensity projections (MIPs) were collected for each hybrid micro-PET/CT mouse 3D image with and without ROIs. The number of ROIs per mouse was counted to generate the number of tumors per mouse; adding the volume of all tumors in each mouse generated tumor burden.

Blood, tumors, spleens, and lungs were collected at the end of the experiment. Tissues were fixed as mentioned before. Neutrophil depletion and changes in tumor and spleen immune cell populations were determined by flow cytometry as explained before. Cell markers used included PE-Cy™5-CD45 (clone 30-F11), Brilliant Violet 711™CD3ε (clone 145-2C11), APC-CD49b (clone DX5), and FITC-CD335 (NKp46) (clone 29A1.4) from BD Biosciences, Brilliant Violet 421™-Ly6G (clone 1A8, BioLegend), Alexa Fluor® 700-CD11b (clone M1/70, eBiosciences), and PE-F4/80 (clone CI:A3-1, AbD Serotec).

Lungs were paraffin-embedded and sectioned at the UWCCC EPL. Neutrophil counts in tumors and lungs were taken to validate flow cytometry data. Immunohistochemical analysis was conducted using anti-Ly6G as previously explained. Slides were imaged at the University of Wisconsin – Madison Laboratory of Computational Instrumentation (LOCI) using the Olympus BX53 histology scope (Olympus, Center Valley, PA, USA). Eight fields per tumor slide were captured with the 40x N.A.: 0.75, UPlanFLN, W.D.: 0.51 objective (Olympus), using Olympus cellSens Standard 1.13 software. Ly6G-positive cells were counted manually for each image and averaged per tumor.

Lungs were also sectioned through every 100 μm and stained with H&E at the UWCCC EPL. Lung metastatic lesions were imaged at 10x N.A.:0.30, UPlanFLN, W.D.:2.1 objective at the University of Wisconsin – Madison LOCI. The area of each lesion in each slide was quantified using the FIJI (ImageJ) (National Institutes of Health, Bethesda, MD, USA) software [30]. The total number of lesions per mouse was counted, and the largest area for each metastatic lesion was collected.

### Statistical analysis

All statistical analysis was conducted using GraphPad Prism 5 (GraphPad Software, La Jolla, CA, USA). The unpaired Student *t* test was used for all experiments to test the differences between means from WT and COL samples. Differences were considered significant when *p* was <0.05. Data are presented as single data points and the mean.

## Results

### COL and WT tumor microenvironments have inherently distinct cytokine expression levels

**Fig. 1:**
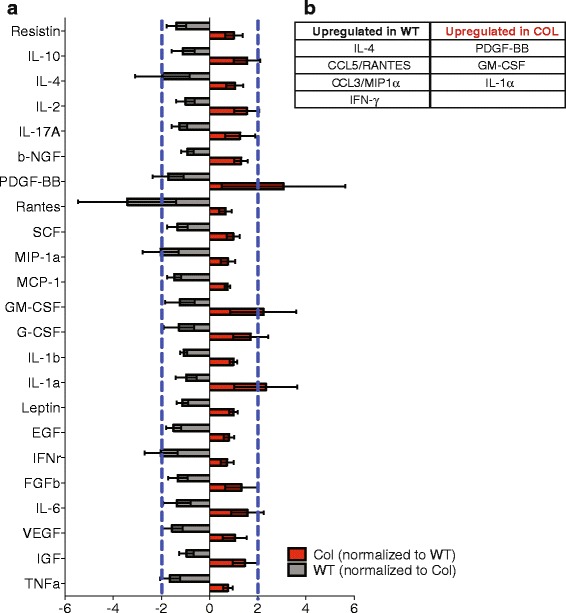
Cytokines in tumor mice expressing the polyoma virus antigen and heterozygote for the collagen-1α chain mutation (*COL*) and wild-type (*WT*) tumor microenvironments are inherently distinct. **a** Cytokine levels from whole WT and COL mammary tumor lysates determined by chemiluminescent Mouse Cytokine ELISA Plate Array. Results are expressed as fold change: WT readings are normalized to COL readings (*gray*) to show cytokines upregulated in WT tumors, and COL readings are normalized to WT readings (*red*) to show cytokines upregulated in COL tumors. *Blue line* denotes a two-fold increase (*n* = 4 independent tumors). **b** Table indicates cytokines with a two-fold increase or greater. *NGF* nerve growth factor, *PDGF* platelet-derived growth factor, *Rantes* regulation on activation, normal T-cell expressed and secreted, *SCF* stem cell factor, *MIP* macrophage inflammatory protein, *MCP* monocyte chemoattractant protein, *GM-CSF* granulocyte monocyte-colony stimulated factor, *EGF* epidermal growth factor, *IFN* interferon, *FGF* fibroblast growth factor, *VEGF* vascular endothelial growth factor, *IGF* insulin-like growth factor

**Fig. 2:**
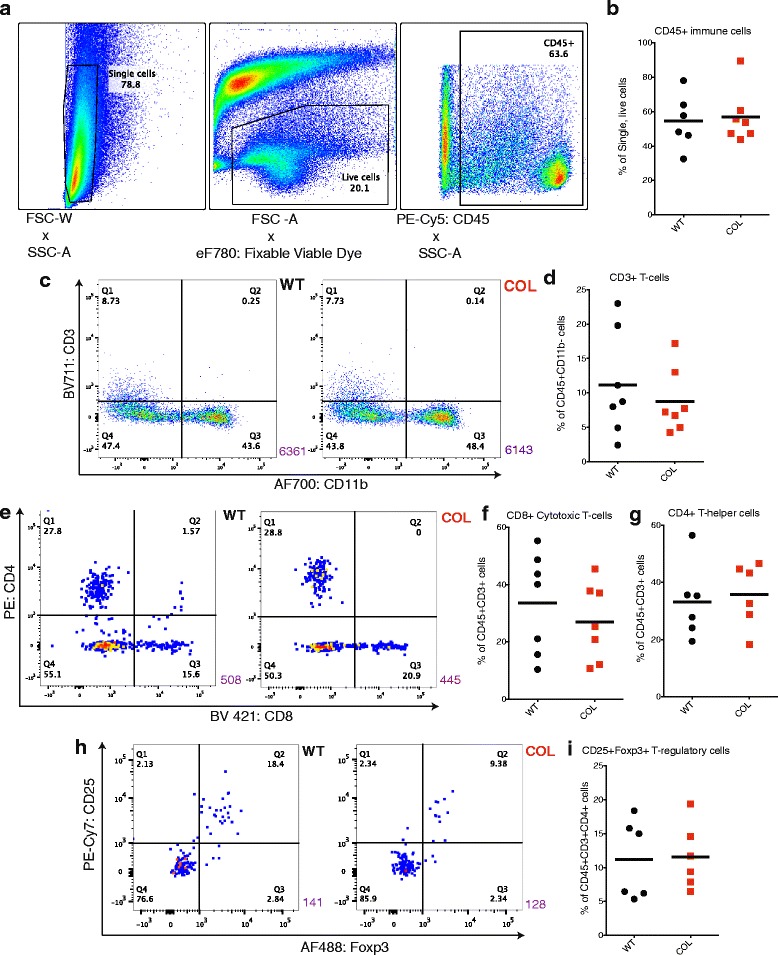
Lymphocyte recruitment is not altered in late-stage collagen-dense tumors. **a** Flow cytometry gates used to determine lymphocytes. **b** Percentage of CD45+ immune cells in wild-type (*WT*) and collagen-dense (*COL*) mammary tumors (*n* = 6). **c** Flow cytometry of CD45+ cells comparing CD3 to CD11b in WT (*left*) and COL (*right*) mammary tumors, and the percentage of CD45+CD3+ T-cells present (*n* = 7) (**d**), CD45+CD3+CD8+ cytotoxic T-cells (**e** and **f**), CD45+CD3+CD4+ helper T-cells (**e** and **g**), and CD45+CD3+CD4+CD25+Foxp3+ regulatory T cells (**h** and **i**) (*n* = 6). *Purple num*ber (*lower right*) represents the number of total events in each graph. These cells were fixed for intracellular staining. *FSC* forward scatter, *SSC* side scatter, *PE* phycoerythrin

We previously reported that the number of PyVT mammary tumors and lung metastases in a COL background is higher than the number of PyVT tumors and lung metastases in a WT background [12]. To characterize whether there is a change in the tumor-promoting COL immune microenvironment, we conducted chemiluminescent ELISA to analyze the expression levels of 23 cytokines found in lysed whole mammary tumors from 15-week-old mice (Fig. 1a, b). Expression of interleukin-4 (IL-4), regulation on activation, normal T cell expressed and secreted (RANTES, CCL5), macrophage inflammatory protein 1α (MIP-1α, CCL3), and Interferon-γ (IFN-γ) was at least two-fold higher in WT tumors compared to COL tumors. IL-4, RANTES, and IFN-γ, are largely involved in T cell signaling, recruitment and activation. In contrast, in COL tumors the expression of platelet-derived growth factor subunit B (PDGF-BB), granulocyte monocyte-colony stimulated factor (GM-CSF), and IL-1α was increased two-fold or more compared to WT tumors (Fig. 2b). Of these, GM-CSF is a chemoattractant for monocytes and neutrophils, and it enhances production and survival of monocytes and granulocytes*.* These data suggest that tumors arising in collagen-dense microenvironments secrete distinct cytokines and may recruit distinct subsets of immune cells compared to tumors in WT mice.

### T lymphocyte recruitment is not altered in the COL tumor microenvironment

**Fig. 3:**
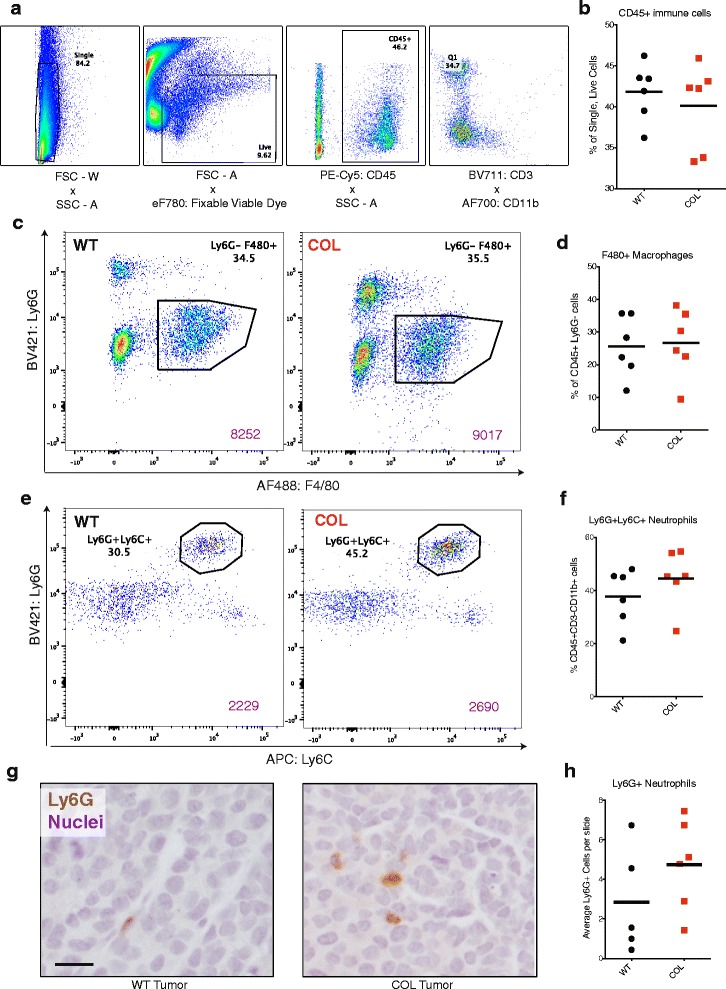
Late-stage collagen-dense mammary tumor recruitment of myeloid cells is not altered. **a** Flow cytometry gates used to determine myeloid cells. **b** Percentage of CD45+ immune cells in wild-type (*WT*) and collagen-dense (*COL*) mammary tumors at 15 weeks (*n* = 6, each of which was an independent pair of age-matched littermate WT and COL mice). **c** Flow cytometry of CD45+CD3– cells comparing Ly6G to F4/80 in WT (*left*) and COL (*right*) mammary tumors. *Purple number* represents the number of total events in each graph. **d** Percentage of CD45+CD3-CD11b+ cells that are F4/80+ macrophages in WT and COL mice. **e** Flow cytometry of CD45+CD11b+CD3– cells comparing Ly6C to Ly6G in WT (*left*) and COL (*right*) mammary tumors; **f** the percentage of CD45+CD3-CD11b+ Ly6G+Ly6C+ neutrophils present. **g** Immunohistochemical analysis of WT (*left*) and COL (*right*) mammary tumors. Tumors were stained with Ly6G (1A8) in diaminobenzidine and counterstained with hematoxylin. *Scare bar* 50 um. **h** average number of Ly6G+ cells found in eight fields of view per tumor slide (*n* = 4)

The cytokine panel suggested that signaling to T cells is greater in WT tumors compared to COL tumors. Differences in lymphocyte recruitment into WT and COL tumors at 15 weeks were determined by flow cytometry with a panel of T cell markers. The percentage of CD45+ leukocytes found in COL tumors did not vary from the percentage found in WT tumors (Fig. 2a, b). Thus, we do not think that COL tumors were harder to dissociate, as we were able to recover a very similar number of immune cells to that recovered from WT tumors. Moreover, we found no significant difference in the percentage of CD45+CD3+CD11b– T cells found in WT and COL tumors (Fig. 2c, d). We also compared CD8 cytotoxic T cell and CD4 helper T cell markers in WT and COL tumors (Fig. 2e), and found that the percentage of CD45+CD3+CD8+ cytotoxic T cells (Fig. 2f) and CD45+CD3+CD4+ helper T-cells (Fig. 3g) did not significantly differ between WT and COL tumors. Recruitment of other lymphocytes such as CD19+ B-cells and NKp46+ natural killer (NK) cells also did not differ in WT versus COL tumors (Additional file 1A and B).

We also quantified CD4+CD25+Foxp3+ regulatory T cells. These cells are characterized by reducing T cell proliferation and cytotoxicity and by contributing to regulatory T cell expansion [31]. When comparing CD25 and Foxp3 regulatory T cell markers (Fig. 2h), the percentage of CD45+CD3+CD4+CD25+Foxp3+ regulatory T cells found in WT and COL tumors was not different (Fig. 2i).

### The overall myeloid cell populations are not altered in a COL tumor microenvironment

The cytokine panel suggested that signaling to monocytes and neutrophils is greater in COL tumors compared to WT tumors. We conducted flow cytometry analysis of cells dissociated and isolated from WT and COL tumors at 15 weeks of age. The percentage of CD45+ leukocytes found in COL tumors did not differ from the percentage found in WT tumors (Fig. 3a, b). When comparing cells expressing Ly6G versus F4/80 in tumors, the percentage of F4/80+ macrophages from the CD45+Ly6G– population did not significantly vary between WT and COL tumors (Fig. 3c, d). Of cells expressing CD45+CD3-CD11b+, we found no significant difference in the percentage of Ly6G+Ly6C+ neutrophils in COL tumors compared to WT (Fig. 3e, f). We validated our flow cytometry results by staining formalin-fixed paraffin-embedded tumor sections from WT and COL tumor mice with anti-Ly6G (1A8) using DAB as the chromogen and hematoxylin as the counterstain (Fig. 3g). Figure 3h shows that the average number of Ly6G+ neutrophils found in eight fields of view was not significantly increased in COL tumors.

Some studies denote CD11b+Ly6G+Ly6C+ myeloid cells as granulocytic myeloid-derived suppressor cells (MDSCs). As there was no change in the number of T cells, specifically regulatory T cells, in WT versus COL tumors, the Ly6G+ cells observed in the mammary tumors were likely not MDSCs. Therefore, we refer to our population of interest as Ly6G+Ly6C+ neutrophils in the context of this study.

### Spleens from COL tumor mice are enlarged, suggesting an advanced disease state

**Fig. 4:**
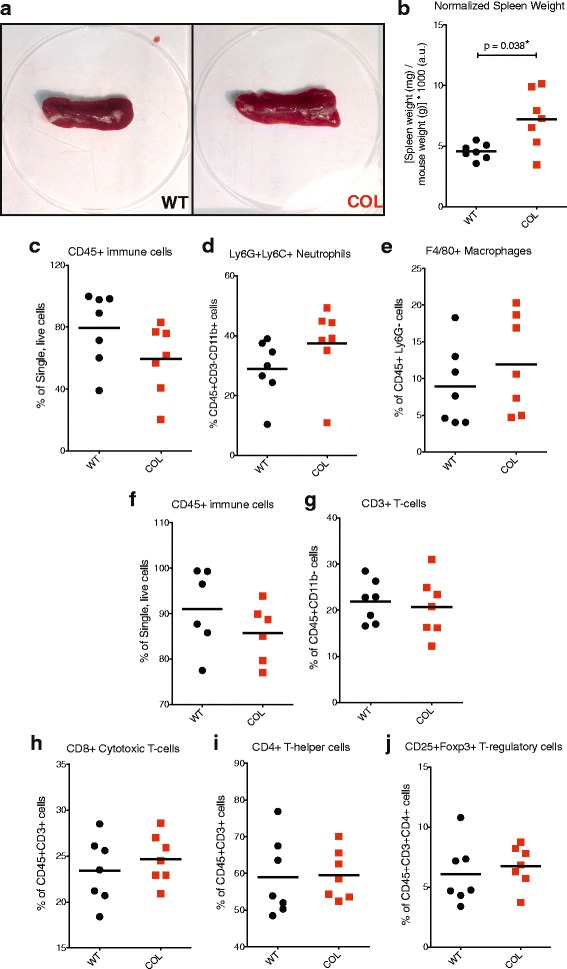
Spleens from late-stage collagen-dense tumor mice are enlarged relative to wild-type (*WT*) mice. **a** Example spleens from WT (*left*) and tumor mice expressing the polyoma virus antigen and heterozygote for the *col1a1* mutation (*COL*) (*right*). Note the increased size of the COL spleens. **b** Spleen weight (mg) normalized to mouse weight (g) from both WT and COL mice (*n* = 7). **c** Percentage of CD45+ immune cells, **d** Ly6G+Ly6C+ neutrophils and **e** F4/80+ macrophages found in WT and COL spleens, determined by flow cytometry as in Fig. 3. Note that there is a trend toward decreased CD45+ immune cells (*p* = 0.13) and increased Ly6G+Ly6C+ neutrophils in COL tumors (*p* = 0.18) (**f**) CD45+ immune cells (lymphoid panel, fixed cells), (**g**) CD3+ T-cells, (**h**) CD8+ cytotoxic T-cells, (**i**) CD4+ T helper cells, and (**j**) Foxp3+ T regulatory cells found in WT and COL spleens, determined by flow cytometry as in Fig. 2)

Splenocytes were isolated to provide controls for flow cytometry experiments. While harvesting the tissue, we observed enlarged spleens in COL tumor mice at 15 weeks of age (Fig. 4a). Spleen weight was measured and normalized to the total weight of each mouse. We found a significant increase in the weight of spleens from COL tumor mice compared to WT tumor mice (Fig. 4b). Characterization of the immune cell profiles in the spleens demonstrated no significant changes in CD45+ immune cells in COL spleens compared to WT spleens (Fig. 4c), whether considering live cells or fixed cells (Fig. 4f). Of the cells expressing CD45+CD11b+, the percent of Ly6G+Ly6C+ neutrophils was not significantly increased in COL spleens compared to WT spleens (Fig. 4d). The percent of F4/80+ macrophages did not differ between COL and WT spleens (Fig. 4e). Using the same lymphocyte flow cytometry cell marker panel as above, we found that the percentage of CD45+CD3+ T cells and T cell subsets: CD8+ cytotoxic T cells, CD4+ helper T cells, and CD4+CD25+Foxp3+ regulatory T cells did not differ in COL spleens compared to WT spleens (Fig. 4h–j). CD19+ B cells and NKp46+ NK cells also did not differ in spleens from WT versus COL tumor mice (Additional file 1C and D). Complete blood counts (Additional file 2A–E), showed a trend towards increased neutrophil to lymphocyte ratio (*p* = 0.06) in COL tumor mice, which is also associated with poor clinical outcome in a population of patients with breast cancer [32]. These results suggest that enlarged spleens and changes in circulating leukocyte counts are whole-body global effects seen in tumor mice with increased collagen.

We did not observe any differences in immune populations due to the *Col1α1* transgene in the absence of PyVT tumors in the mammary glands (do not express the PyVT antigen) (Additional file 3A–E). The spleens of normal, non-tumor-bearing COL mice were not enlarged (Additional file 4A), their immune cell population recruitment did not differ significantly (Additional file 4B–F), and there were no significant changes in circulating blood leukocyte counts (Additional file 5A–E).

### Depleting neutrophils with anti-Ly6G reduces tumor burden of COL mice

**Fig. 5:**
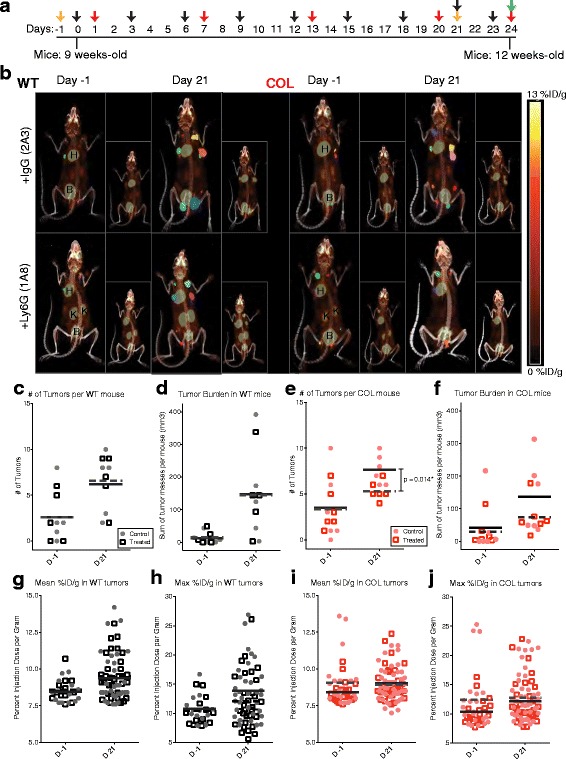
Neutrophil depletion with anti-Ly6G antibody reduces collagen-dense tumor formation as measured by ^18^FDG-PET glucose uptake. **a** Experimental time line showing timing of injections of 5.5 μg/g of control IgG (2A3) or Ly6G (1A8) into the peritoneal cavity of wild-type (*WT*) tumor mice and tumor mice expressing the polyoma virus antigen and heterozygote for the *col1a1* mutation (*COL*) mice (*n* = 5–6 in each group) at 9 weeks of age for 24 days. *Yellow arrows* indicate day of 2’-deoxy-2’-[^18^F]fluoro-D-glucose (^18^FDG) hybrid positron emission tomography (PET)-computed tomography (CT) imaging. *Black arrows* indicate day of treatment. *Red arrows* indicate day of blood collection. *Green arrow* indicates day of tissue collection (tumors, spleens, and lungs). **b** Detection of mammary tumors by ^18^FDG-PET/CT. Coronal sections of co-registered ^18^FDG-PET and CT images of WT and COL tumor mice before treatment at 9 weeks of age (*Day −1*) and after treatment at 12 weeks of age (*Day 21*). Regions of interest are drawn and colored at the mouse mammary glands with visible tumors. Physiologic tracer uptake in heart (*H*), kidneys (*K*), and bladder (*B*) is marked. Total number of tumors in **c** WT mice and **e** COL mice as determined by ^18^FDG-PET at day −1 (*D –1*) and day 21 *(D 21)*. Sum of the mass (mm^3^) of all tumors per WT (**d**) and COL mouse (**f**) at day −1 and day 21 (*n* = 5–6; each data point equals one mouse). Note that there is a trend toward decreased tumor burden in anti-Ly6G-treated COL tumors (*p* = 0.24). **g** Mean glucose uptake (percent injection dose per gram (%ID/g) in WT and COL (**i**) tumors at day −1 and day 21. **h** Maximum glucose uptake (%ID/g)) in WT and **j** COL tumors at day −1 and day 21 (*n* = 32–46; each data point equals one tumor)

The cytokine array results suggest there may be differences in activation of neutrophils in COL mice, even if the number of neutrophils recruited is not significantly different. The function of Ly6G+ neutrophils in tumor progression was studied by blocking the recruitment of neutrophils in COL and WT tumor mice. Mice were treated with anti-Ly6G or anti-IgG for 24 days starting at 9 weeks of age (Fig. 5a). In order to dynamically monitor tumor growth in this transgenic (i.e., not luciferase-labeled) model, we made use of hybrid micro PET-CT 3D imaging using the uptake of ^18^FDG in WT and COL, control, and Ly6G-depleted mice. Figure 5b shows a representative maximum intensity projection from each group, where ROIs are drawn to indicate where the tumor is and its relative size. As expected, on the day before treatment started (day −1), the number of tumors was the same in both control and treatment groups (Fig. 5c, e). After 21 days, the tumor size increased due to disease progression in the PyVT model in both the WT and COL control arms. Antibody-depletion of Ly6G+ neutrophils significantly slowed the progression of cancer by reducing the number of COL tumors, but not the number of WT tumors after 21 days (Fig. 5c, e). The total tumor burden in WT tumor mice increased over time and was not attenuated by treatment with anti-Ly6G (Fig. 5d). In contrast, the tumor burden decreased with anti-Ly6G treatment in COL mice after 21 days (Fig. 5f). Figure 5g–j displays the mean and maximum percent injection dose per gram of each tumor, and demonstrates that ^18^FDG uptake increases with progression of the disease, but does not change with neutrophil depletion in WT nor COL tumors. PET imaging is a non-invasive imaging modality that utilizes tumor-linked increase in glucose uptake and metabolism to image mammary tumors [33, 34]. Our results show that glucose uptake does not change with treatment or the collagen density of tumors, and therefore we conclude that the changes seen in the number of tumors and tumor volume is a direct effect of blocking Ly6G+ neutrophils.

**Fig. 6:**
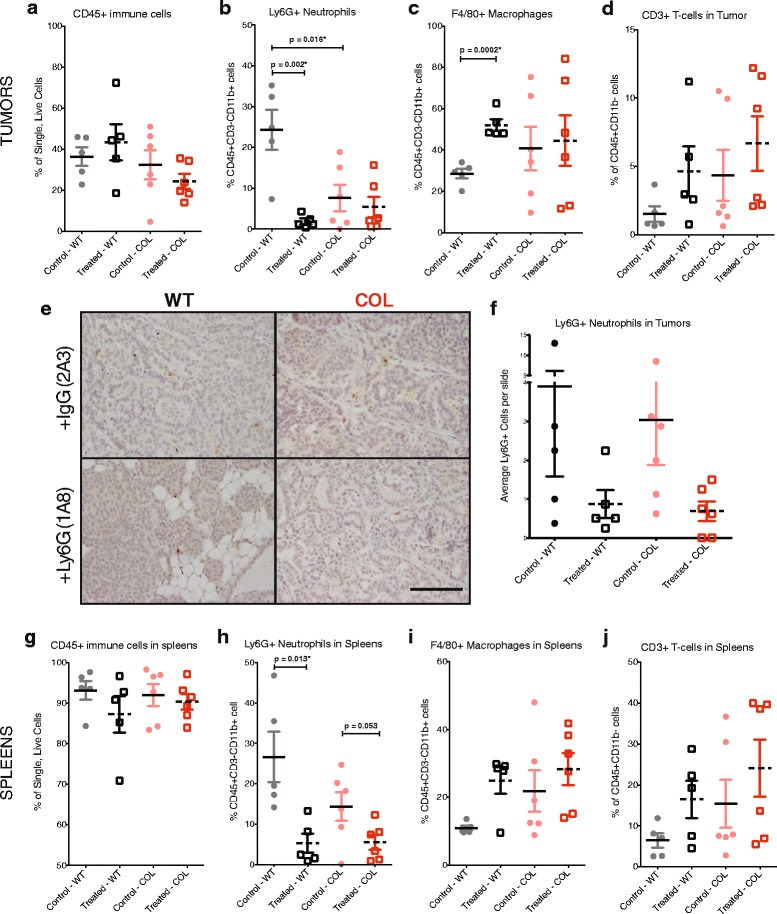
Immune cell recruitment to tumors and spleens treated with anti-Ly6G. Percentage of CD45+ immune cells (**a**), Ly6G+ neutrophils (**b**), F4/80+ macrophages (**c**), and CD3+ T cells (**d**) in wild-type (WT) and collagen-dense (COL) mammary tumors from 12-week-old mice treated with control IgG or Ly6G (*n* = 5–6) obtained by flow cytometry at day 24 of the treatment regimen (described in Fig. 5a). Immunohistochemistry of tumors from the four treatment groups (**e**). Tumors were stained with Ly6G (1A8) in DAB and counterstained with hematoxylin. Scare bar = 100 um. Average number of Ly6G+ cells found in eight fields of view per tumor slide (*n* = 3) (**f**). Percentage of CD45+ immune cells (**g**), Ly6G+ neutrophils (**h**), F4/80+ macrophages (**i**), and CD3+ T-cells (**j**) in spleens of the same treated and untreated WT and COL mice, as shown in **a**–**d**

Tumors isolated from control and treated groups were analyzed by flow cytometry to understand the effect of depleting neutrophils on immune cell recruitment. Figure 6a and g shows that the percentage of CD45+ immune cells was similar in all groups of both tumors and spleens. To evaluate depletion of neutrophils, we quantified the percentage of Ly6G+ neutrophils from immune cells that were CD45+CD3-CD11b+ (Fig. 6b, h). At 12 weeks, anti-Ly6G treatment decreased neutrophil numbers in both WT and COL tumors. Neutrophil depletion was confirmed in spleens of WT and COL tumor mice, demonstrating a significant depletion in Ly6G+ neutrophils with antibody treatment (Fig. 6h). Interestingly, treatment with anti-Ly6G significantly increased the percentage of F4/80+ macrophages in WT tumors, but not in COL tumors, which had variable numbers of F4/80+ macrophages in both control and treatment arms (Fig. 6c). Data from spleens also showed the trend of increased F4/80+ macrophages in treated WT spleens. (Fig. 6i). Changes in the number of NK cells were not observed in tumors and spleens (Additional file 6A and C). No spleen enlargement occurred upon anti-Ly6G treatment at 12 weeks (Additional file 6B). Nor were there other global effects due to anti-Ly6G treatment, such as changes in the number of circulating blood cells (Additional file 7A–E).

### Depleting neutrophils with anti-Ly6G reduces the number of lung metastases in COL but not WT mice

**Fig. 7:**
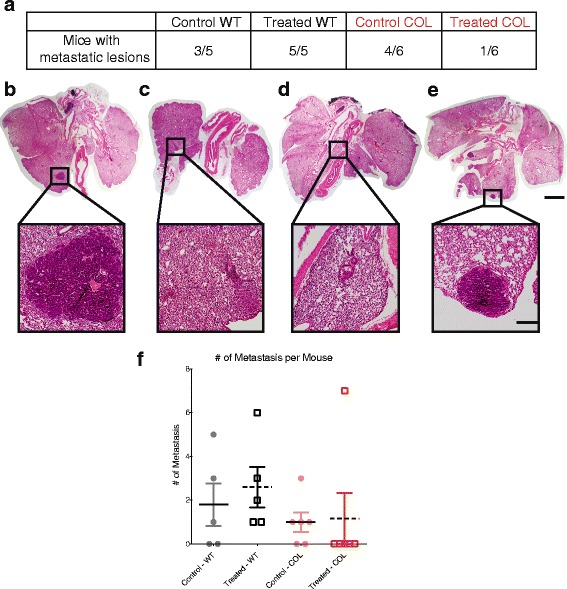
Neutrophil depletion with anti-Ly6G antibody reduces the number of dense-collagen metastatic lesions. (**a**) Numbers of mice with metastasis per group. Note that all treated wild-type (*WT*) tumor mice developed metastatic lesions versus one tumor mouse expressing the polyoma virus antigen and heterozygote for the *col1a1* mutation (*COL*) (*p* < 0.05). Representative images of lungs with corresponding metastasis in the WT control group (**b**), WT anti-Ly6G treated group (**c**), COL control group (**d**), and COL anti-Ly6G-treated group (**e**). *Scale bar* 2 mm (*whole lung image*) and 250 μm (*metastatic lesion image*). **f** Quantification of the number of metastatic lesions found in each mouse (*n* = 5–6 per group)

Lungs from WT and COL mice from both control and Ly6G-depleted arms were sectioned and the number of metastatic lesions throughout the whole lung determined. Anti-Ly6G-treated COL mice had an 80 % response to treatment; only one out of six mice had lung metastases. In contrast, all Ly6G-depleted WT mice developed metastatic lesions (Fig. 7a). In fact, treatment with anti-Ly6G increased the number of WT animals with metastases and the total number of metastases (Fig. 7b–f). The number of Ly6G+ neutrophils in the lungs was quantified by immunohistochemical analysis (Additional file 8A and B). There was no correlation between the numbers of Ly6G+ neutrophils found in the lungs and the number of lung metastatic lesions (Additional file 8C).

## Discussion

Although mammographic density is associated with increased risk of developing breast cancer, the mechanisms underlying this increased risk are poorly understood. High mammographic density is associated with changes in the stroma, including an increase in collagen 1 [8–10]. Here we use the COL MMTV-PyVT model, which has increased collagen deposition, a higher tumor incidence, and more metastases compared to tumors in WT animals [12]. Now, we seek to further characterize the mechanisms by which increased stromal collagen enhances tumor progression by identifying immune cell populations present in the COL tumor microenvironment and their effect on tumor progression. The pro-tumor progression role of leukocyte infiltrates has been shown in the MMTV-PyVT mammary tumor model, but not in COL mammary tumors [35–37]. The expression of cytokines GM-CSF, IL-1α, and PDGF-BB, is upregulated in tumors from COL mice, while IL-4, MIP-1a, RANTES, and IFN-γ expression is increased in tumors from WT mice. These differences suggest there is an altered host response for tumors arising in collagen-dense microenvironments.

Despite differences in cytokine profiles, we did not see a significant change in the recruitment of distinct immune cell populations comparing WT to COL tumors. Rather, there was a functional difference in the role of neutrophils, as depletion of Ly6G+ neutrophils with anti-Ly6G (1A8) antibody diminishes tumor progression specifically in COL mice but has little effect on WT Ly6G-depleted tumors. Most importantly, depletion of neutrophils with anti-Ly6G significantly inhibited lung metastasis in COL mice, but did not diminish metastases in WT mice. Thus, we found that neutrophils have a tumor promoting effect specifically in the context of COL mammary tumors, but do not significantly contribute to progression and metastasis in WT tumors.

Others have also performed similar neutrophil depleting studies using anti-Ly6G (1A8) in orthotopic models [23, 28, 38, 39], but these studies did not investigate the role of collagen density. These studies present conflicting evidence on whether neutrophils play a role in breast cancer metastasis. While treating orthotopic 66c14 mouse mammary tumors with anti-Ly6G decreases lung metastasis [28], depletion of neutrophils in orthotopic MMTV-PyVT or 4T1 mouse mammary carcinoma models does not affect primary tumor size and results in increased lung metastasis [27]. Tabariés et al. recently demonstrated that Ly6G+ granulocytes are recruited to lung and liver metastases in the 4T1 model. In their study, depleting neutrophils with anti-Ly6G did not affect lung metastasis, but it did reduce liver metastasis [23]. All of these models rely on cell lines or surgical procedures for the creation of tumors and metastases. An intriguing possibility is that the conflicting results may be due to different wound-healing and fibrotic responses in these different studies, although changes in the ECM were not determined. Here, we used the MMTV-PyVT model, which develops spontaneous metastasis starting after 10 weeks of age [40] and does not require surgical intervention that may confound the results with variable wound-healing effects. In the current study, anti-Ly6G treatment had no effect on lung metastases in WT mice, but significantly decreased metastases in COL mice.

Our flow cytometry analysis of immune cell types showed an increase in the number of CD11b+F4/80+ macrophages in treated WT tumors. Because TAMs have an established role in tumor progression and metastasis [19, 41], the increase in macrophages may carry on with invasion and metastasis in the MMTV-PyVT tumors even after depletion of Ly6G+ cells in WT mice. A role for macrophages in metastasis following Ly6G depletion is supported by our observation that the only individual mouse with metastasis in the treated COL group also had the largest recruitment of CD11b+F4/80+ macrophages (data not shown). In the COL mouse model collagen I is increased throughout the whole animal. We hypothesized that COL lungs can also recruit Ly6G+ neutrophils, but our results did not support this hypothesis, because the number of lung metastatic lesions did not correlate with the number of Ly6G+ neutrophils found near the lung metastases.

It is not entirely clear why depleting Ly6G+ neutrophils slows tumor growth in COL mice but not WT mice. This finding demonstrates that Ly6G+ neutrophils have an important role in COL tumor progression, but not in WT tumor progression. Possibly the Ly6G+ neutrophils in the COL tumors are polarized towards a pro-tumor phenotype compared to the Ly6G+ neutrophils in WT tumors. If Ly6G+ neutrophils in WT tumors are not pro-tumorigenic, depleting them would have little effect, which is what we observed. The idea that there is a fundamental difference in the host response in the COL versus the WT tumor microenvironment is consistent with our finding that there are differences in the cytokines released within COL compared to WT tumors.

Studies of mesothelioma and lung carcinoma lines injected into the flank of mice have been used to demonstrate the polarization of neutrophils, identifying a tumor-associated neutrophil (TAN) population analogous to TAMs. TANs can have anti-tumorigenic (N1) or pro-tumorigenic (N2) phenotypes, where the predominant N2 phenotype is driven by the presence of transforming growth factor (TGF)-β [38]. These models also show that TANs at early stages of tumor growth are more cytotoxic to tumor cells by either directly killing tumor cells or by secreting reactive oxygen species (ROS), whereas later in tumor development, they acquire a more tumor-supportive phenotype [39]. In pancreatic cancer, neutrophils residing within the tumors express MMP-9, which then liberates vascular endothelial growth factor (VEGF) and promotes angiogenesis [42]. Ly6G+ neutrophils are recruited preferentially to liver metastases in the 4T1 model, where they are alternatively polarized towards an N2, pro-tumorigenic phenotype [23]. Several studies reveal that N2 neutrophils express MMP9, NE, and *CXCR4,* among other pro-tumor mediators [43–45]. We have found no evidence that the collagen-dense tumor microenvironment facilitates the expression of these markers in Ly6G+ neutrophils when compared to neutrophils in non-dense tumors (data not shown). Our results are consistent with a more general notion that the cell-ECM interactions occurring in the collagen-dense microenvironment are associated with a neutrophil-dependent tumor progression and metastatic process.

In vitro studies of neutrophil biology have demonstrated the ability of neutrophils to migrate in 3D collagen I matrices [46]. Neutrophils can also adhere and become activated in type I collagen [47]. Peptides derived from collagen breakdown are chemotactic for neutrophils in vivo and in vitro [48]. These neutrophil capabilities, especially neutrophil migration in response to chemoattractants, involve integrin-β2, integrin-α2, and integrin-β1. The two latter integrins mediate cell adhesion to collagen I fibrils [49, 50]. Although rapid leukocyte migration in response to chemokines can be integrin-independent [51], neutrophil chemotaxis in collagen I is also mediated by the collagen receptor, discoidin domain receptor 2 (DDR2). DDR2 regulates neutrophil directionality and persistence by releasing MMPs (MMP-8) that cleave collagen [52]. These studies support the recruitment of neutrophils to COL microenvironments in vivo.

Our data suggest that tumors arising in a collagen-dense microenvironment are fundamentally different from those arising in a non-dense microenvironment, as there are key differences in cytokine signals that support tumor progression. All cytokines have specific roles in wound healing or infection, but once the immune system is challenged with cancer, some of these functions are co-opted by the tumor. The presence and function of Ly6G+Ly6C+ neutrophils in COL mammary tumors is likely supported by increased GM-CSF levels compared to WT tumors. This proinflammatory cytokine stimulates proliferation and induces maturation of granulocyte and monocyte progenitor cells. GM-CSF has been described as a strong chemoattractant for mouse and human neutrophils [53, 54] that can differentiate monocytes into dendritic cells and polarize T cells into a T helper-1 (Th-1) phenotype [55, 56]. GM-CSF can block the angiogenic functions of tumor-associated macrophages in FVB mice orthotopically injected with PyVT tumors [57]. Moreover, GM-CSF is associated with aggressive breast cancer subtypes. GM-CSF secreted by cells derived from the FVB202 mouse model of breast cancer with inactivated neu (*Erbb2*) can recruit and aid in survival and differentiation of CD11b+GR-1+ cells, which include CD11b+Ly6G+ cells [58]. GM-CSF secreted by human breast cancer cells primes plasmacytoid pre-dendritic cells (pDC) to polarize the immune microenvironment toward a regulatory Th-2 phenotype [59]. Homodimer PDGF-BB is also upregulated in COL tumors. High levels of PDGF-B lead to over-activity of tyrosine kinase PDGF receptor and tumor cell proliferation. This mitogen stimulates growth, survival, and migration of mesenchymal/stromal cells [60]. Breast cancer cell-secreted PDGF targets stromal cells and initiates tumor desmoplasia [61]. In vitro, PDGF-BB is a chemoattractant for neutrophils and monocytes, and can activate human neutrophils [62, 63]. Thus, our finding of elevated PDGF-BB in a dense collagen microenvironment is consistent with an increase in neutrophil activation. Il-1α is not commonly found in the circulation except in severe disease. IL-1α can induce matrix degradation and remodeling by breast cancer fibroblasts. Breast cancer cells can secrete IL-6 and IL-8 in response to IL-1α [64]. Although not part of the cytokine array, IL-8 (KC, MIP-2, and LIX homologues in mouse) is a major neutrophil chemoattractant [65, 66]. Our data suggest that all of these contribute to the progression of mammary cancer in the COL tumor microenvironment through the interplay of different components of the tumor microenvironment (collagen, cancer cells, fibroblasts, and neutrophils). It has yet to be determined exactly which cell type contributes to the increased production of these cytokines in this model. Tumor cell cytokine secretion has been widely studied in vitro, but new techniques must be developed to answer these questions in a complex, in vivo COL tumor microenvironment.

Of the signals downregulated in collagen -dense mammary tumors, CCL5 is the chemokine that has the greatest differential expression in WT mammary tumors. It is produced by activated T cells and is a strong T cell chemoattractant. The role of CCL5 in breast cancer progression and metastasis has been established: this includes upregulation of MMP secretion, pro-tumor effects by generating MDSC in the bone marrow, and a reduction in metastasis when low levels of CCL5 are expressed [67–70]. Also upregulated in WT tumors, IL-4 is one of the most studied Th-2 cytokines [71]. Recent studies of IL-4/IL-4 receptor (IL-4R) signaling in breast cancer cells in vitro and in vivo show enhanced cell survival and proliferation via signal transducer and activator of transcription (STAT)/protein kinase B (AKT)/mitogen-activated protein kinase (MAPK) signaling. It is also produced by activated T cells and promotes differentiation, survival, and proliferation of B and T lymphocytes via IL4R signaling [72].

T cells (CD3+ T lymphocytes, especially CD8+ T cells) have also been shown to respond to the potent chemoattractant, CCL3 [73]. CCL3 can recruit and activate monocytes and is involved in anti-tumor activities as a Th-1 cytokine and increasing NK cell cytotoxicity [74]. On the other hand, CCL3 has been implicated in promoting breast cancer metastasis to the lungs [75]. Also a Th-1 cytokine, IFN-γ works with T lymphocytes to regulate tumor cell immunogenicity by preventing the development of spontaneous epithelial carcinoma [76]. The presence of these cytokines only in WT tumors leads to the hypothesis that COL tumors have reduced T cell signaling (recruitment and activation), which may account for their increased tumor incidence and metastasis. We found no major differences in the recruitment of T cells and subgroups in WT versus COL mammary tumors and spleens. B cell and NK cell populations also did not differ in recruitment. These results open the doors to studies of lymphocyte activation, proliferation, and cytotoxicity in either 3D in vitro collagen gels or in the MMTV-PyVT collagen-dense model.

## Conclusions

We found that MMTV-PyVT tumors arising in a dense collagen microenvironment have altered cytokine expression compared to tumors arising in a non-dense microenvironment. Cytokines involved in neutrophil maturation and recruitment, including GM-CSF, PGDF-BB, and IL-1α, were altered in dense-collagen tumors. Depletion of Ly6G+ neutrophils diminished tumor formation, tumor burden, and lung metastasis, specifically in collagen-dense mammary tumors. Identifying neutrophils as key players in COL mammary tumor progression has important clinical implications for patient populations with mammographically dense breast tissue. Although more research is necessary, tumor-associated neutrophils may be useful prognostic markers and possible targets for breast cancer therapy, especially for patients with dense breast tissue.

## Additional files

Additional file 1: Quantification of B cell and NK cells found in late-stage mammary tumors. **A** The percentage of CD45+CD3- cells that are CD19+ B cells in WT and COL tumors and **C** spleens at 15 weeks. **B** The percentage of CD45+CD3- cells that are Nkp46+ NK cells in WT and COL tumors and **D** spleens at 15 weeks. (PDF 293 kb) Additional file 2: Tumor mice complete blood counts at late tumor stages. **A** White blood cell, **B** lymphocyte, **C** monocyte, and **D** neutrophil counts in 10^3^ per μl of blood from WT and COL tumor mice at 15 weeks. Blood was obtained by a retro-orbital bleeding procedure and counted on a Hemavet 950FS hematology system. **E** Neutrophil to lymphocyte ratio (NLR) a.u. *Green dash line* denotes no change (NLR = 1) (*n* = 5). (PDF 311 kb) Additional file 3: Quantification of immune cells in wild-type and collagen-dense non-tumor mammary glands. **A** Percentage of CD45+ immune cells in WT and COL mammary glands at 15 weeks (*n* = 3, of which each is an independent pair of age-matched littermate WT and COL non-tumor mice). **B** Percentage of CD45+CD3-CD11b+ cells that are F4/80+ macrophages in mammary glands. **C** Percentage of CD45+CD3-CD11b+ cells that are Ly6G+Ly6C+ neutrophils. **D** Percentage of CD45+CD3- cells that are Nkp46+ NK cells. **E** Percentage of CD45+CD3+ T-cells in mammary glands, determined by flow cytometry as in Fig. 1. (PDF 313 kb) Additional file 4: Quantification of immune cells in spleens from wild-type and collagen-dense non-tumor mice. **A** Spleen weight (mg) normalized to mouse weight (g) from both WT and COL mice (*n* = 3). **B** Percentage of CD45+ immune cells, **C** CD45+CD3-CD11b+ cells that are F4/80+, **D** CD45+CD3-CD11b+ cells that are Ly6G+Ly6C+ neutrophils, **E** CD45+CD3-CD11b+ cells that are Nkp46+ NK cells, and **F** CD45+CD3+ T cells found in WT and COL spleens from non-tumor mice at 15 weeks, determined by flow cytometry as in Additional file 3. (PDF 327 kb) Additional file 5: Non-tumor mice complete blood counts at 15 weeks of age. **A** White blood cell, **B** lymphocyte, **C** monocyte, and **D** neutrophil counts in 10^3^ per μl of blood from WT and COL non-tumor. Blood was obtained as in Additional file 2. **E** Neutrophil to lymphocyte ratio (NLR) a.u. *Green dash line* denotes no change (NLR = 1) (*n* = 3). (PDF 304 kb) Additional file 6: Recruitment NK cells in neutrophil-depleted tissues. **A** The percentage of CD45+CD3– cells that are Nkp46+ NK cells in anti-Ly6G treated or IgG control WT and COL tumors at 12 weeks. **B** Spleen weight (mg) normalized to mouse weight (g) from anti-Ly6G treated and IgG control WT and COL tumor mice. **C** The percentage of CD45+CD3– cells that are Nkp46+ NK cells in anti-Ly6G treated or IgG control WT and COL spleens at 12 weeks. (PDF 300 kb) Additional file 7: Complete blood counts at different time points during neutrophil depleting study. **A** White blood cell, **B** monocyte, **C** granulocyte, and **D** lymphocyte count in 10^3^ per μl from treated and control WT and COL mice obtained by saphenous vein bleeding, and counted on a Hemavet 950FS hematology system. **E** Neutrophil to lymphocyte ratio (NLR) a.u. (*n* =5 or 6). (PDF 336 kb) Additional file 8: Neutrophil counts in metastatic lungs do not correlate with number of metastases in depletion study. **A** Immunohistochemical analysis of lungs from the four treatment groups. Lungs were stained with Ly6G (1A8) in DAB and counterstained with hematoxylin. *Scare bar* 100 um. **B** Average number of Ly6G+ cells found in eight fields of view per slide (*n* = 3). **C** Average number of Ly6G+ cells per lung versus the number of metastatic lesions per lungs. (JPG 558 kb)

## Abbreviations

APC: allophycocyanin
ABC: avidin-biotin complex
BSA: bovine serum albumin
CCL3: chemokine (C-C motif) ligand 3
CCL5: chemokine (C-C motif) ligand 5
COL: tumor mice expressing the polyoma virus antigen and heterozygote for the collagen-1α chain mutation
Col1α1: type I collagen-α1
CT: computed tomography
DAB: diaminobenzidine
DDR2: discoidin domain receptor 2
DMEM: Dulbecco’s modified Eagle’s medium
DNAse: deoxyribonuclease
ECM: extracellular matrix
EDTA: ethylenediaminetetraacetic acid
ELISA: enzyme-linked immunosorbent assay
EPL: University of Wisconsin Carbone Cancer Center Experimental Pathology Laboratory
FBS: fetal bovine serum
FDG: 2’-deoxy-2’-[^18^F]fluoro-D-glucose
FITC: fluorescein isothiocyanate
FMO: fluorescent minus one
FVB: Friend virus B type
FGF: fibroblast growth factor
GM-CSF: granulocyte monocyte-colony stimulated factor
H&E: hematoxylin and eosin
HRP: horseradish peroxidase
IFN-γ: interferon gamma
IL: interleukin
JAK: Janus kinase
LOCI: University of Wisconsin – Madison Laboratory of Computational Instrumentation
MAPK: mitogen-activated protein kinase
MDSC: myeloid derived suppressor cells
MFI: medium fluorescent intensity
MIP: maximum intensity projection
MIP-1α: macrophage inflammatory protein-1α
MMP: matrix metalloproteinase
MMTV-PyVT: mouse mammary tumor virus - polyoma virus middle T antigen
N1: anti-tumor neutrophil
N2: pro-tumor neutrophil
NE: neutrophil elastase
NK cell: natural killer cell
NRL: neutrophil to lymphocyte ratio
OSEM: ordered-subset expectation maximization
PCR: polymerase chain reaction
pDC: plasmacytoid predendritic cells
PDGF-BB: platelet-derived growth factor subunit B homodimer
PE: phycoerythrin
PET: positron emission tomography
RANTES: regulation on activation, normal T-cell expressed and secreted
ROI: region of interest
ROS: reactive oxygen species
STAT: signal transducer and activator of transcription
TAM: tumor-associated macrophage
TAN: tumor-associated neutrophil
TBS: Tris-buffered saline
TGF: transforming growth factor
TNF: tumor necrosis factor
UWCCC: University of Wisconsin Carbone Cancer Center
VEGF: vascular endothelial growth factor
WT: tumor mice expressing the polyoma virus antigen, but negative for the collagen-1α chain mutation

## References

[1] Boyd NF, Lockwood GA, Byng JW, Tritchler DL, Yaffe MJ (1998). Mammographic densities and breast cancer risk. Cancer Epidemiol Biomarkers Prev.

[2] Boyd NF, Guo H, Martin LJ, Sun L, Stone J, Fishell E (2007). Mammographic density and the risk and detection of breast cancer. N Engl J Med.

[3] Provenzano PP, Inman DR, Eliceiri KW, Keely PJ (2009). Matrix density-induced mechanoregulation of breast cell phenotype, signaling and gene expression through a FAK-ERK linkage. Oncogene.

[4] Zhang K, Corsa CA, Ponik SM, Prior JL, Piwnica-Worms D, Eliceiri KW (2013). The collagen receptor discoidin domain receptor 2 stabilizes SNAIL1 to facilitate breast cancer metastasis. Nat Cell Biol.

[5] Pang J-MBM, Byrne DJ, Takano EA, Jene N, Petelin L, McKinley J (2015). Breast tissue composition and immunophenotype and its relationship with mammographic density in women at high risk of breast cancer. PloS one.

[6] Sun X, Gierach GL, Sandhu R, Williams T, Midkiff BR, Lissowska J (2013). Relationship of mammographic density and gene expression: analysis of normal breast tissue surrounding breast cancer. Clin Cancer Res.

[7] Lin SJ, Cawson J, Hill P, Haviv I, Jenkins M, Hopper JL (2011). Image-guided sampling reveals increased stroma and lower glandular complexity in mammographically dense breast tissue. Breast Cancer Res Treat.

[8] Alowami S, Troup S, Al-Haddad S, Kirkpatrick I, Watson PH (2003). Mammographic density is related to stroma and stromal proteoglycan expression. Breast Cancer Res..

[9] Guo YP, Martin LJ, Hanna W, Banerjee D, Miller N (2001). Growth factors and stromal matrix proteins associated with mammographic densities. Cancer Epidemiol Biomarkers Prev..

[10] Huo CW, Chew G, Hill P, Huang D, Ingman W, Hodson L (2015). High mammographic density is associated with an increase in stromal collagen and immune cells within the mammary epithelium. Breast Cancer Res..

[11] McConnell JC, O'Connell OV, Brennan K, Weiping L, Howe M, Joseph L (2016). Increased peri-ductal collagen micro-organization may contribute to raised mammographic density. Breast Cancer Res.

[12] Provenzano PP, Inman DR, Eliceiri KW, Knittel JG, Yan L, Rueden CT (2008). Collagen density promotes mammary tumor initiation and progression. BMC Med..

[13] Lin EY, Jones JG, Li P, Zhu L, Whitney KD, Muller WJ (2003). Progression to malignancy in the polyoma middle T oncoprotein mouse breast cancer model provides a reliable model for human diseases. Am J Pathol.

[14] Guy CT, Cardiff RD, Muller WJ (1992). Induction of mammary tumors by expression of polyomavirus middle T oncogene: a transgenic mouse model for metastatic disease. Mol Cell Biol.

[15] DeNardo DG, Coussens LM (2007). Inflammation and breast cancer. Balancing immune response: crosstalk between adaptive and innate immune cells during breast cancer progression. Breast Cancer Res.

[16] Dunn GP, Old LJ, Schreiber RD (2004). The immunobiology of cancer immunosurveillance and immunoediting. Immunity.

[17] Condeelis J, Pollard JW (2006). Macrophages: obligate partners for tumor cell migration, invasion, and metastasis. Cell..

[18] Russell DL, Adrian LH (2002). Tumor-associated macrophages in breast cancer. J Mammary Gland Biol Neoplasia.

[19] Qian B, Deng Y, Im JH, Muschel RJ, Zou Y, Li J (2009). A distinct macrophage population mediates metastatic breast cancer cell extravasation, establishment and growth. PLoS One.

[20] Pollard JW (2008). Macrophages define the invasive microenvironment in breast cancer. J Leukoc Biol.

[21] Swierczak A, Mouchemore KA, Hamilton JA, Anderson RL (2015). Neutrophils: important contributors to tumor progression and metastasis. Cancer Metastasis Rev..

[22] Yan HH, Pickup M, Pang Y, Gorska AE, Li Z, Chytil A (2010). Gr-1+ CD11b + myeloid cells tip the balance of immune protection to tumor promotion in the premetastatic lung. Cancer Res.

[23] Tabariès S, Ouellet V, Hsu BE, Annis MG, Rose AA, Meunier L (2015). Granulocytic immune infiltrates are essential for the efficient formation of breast cancer liver metastases. Breast Cancer Res..

[24] Fridlender ZG, Albelda SM, Granot Z (2015). Promoting metastasis: neutrophils and T cells join forces. Cell Res.

[25] Bekes EM, Schweighofer B, Kupriyanova TA, Zajac E, Ardi VC, Quigley JP (2011). Tumor-recruited neutrophils and neutrophil TIMP-free MMP-9 regulate coordinately the levels of tumor angiogenesis and efficiency of malignant cell intravasation. Am J Pathol.

[26] Kitamura T, Qian B-ZZ, Pollard JW (2015). Immune cell promotion of metastasis. Nat Rev Immunol.

[27] Granot Z, Henke E, Comen EA, King TA, Norton L, Benezra R (2011). Tumor entrained neutrophils inhibit seeding in the premetastatic lung. Cancer Cell.

[28] Kowanetz M, Wu X, Lee J, Tan M (2010). Granulocyte-colony stimulating factor promotes lung metastasis through mobilization of Ly6G+ Ly6C+ granulocytes. Proc Natl Acad Sci.

[29] Liu X, Wu H, Byrne M, Jeffrey J, Krane S, Jaenisch R (1995). A targeted mutation at the known collagenase cleavage site in mouse type I collagen impairs tissue remodeling. J Cell Biol.

[30] Schindelin J, Arganda-Carreras I, Frise E, Kaynig V, Longair M, Pietzsch T (2012). Fiji: an open-source platform for biological-image analysis. Nat Methods.

[31] Brandau S, Moses K, Lang S (2013). The kinship of neutrophils and granulocytic myeloid-derived suppressor cells in cancer: Cousins, siblings or twins?. Semin Cancer Biol.

[32] Chen J, Deng Q, Pan Y, He B, Ying H, Sun H (2015). Prognostic value of neutrophil-to-lymphocyte ratio in breast cancer. FEBS Open Bio..

[33] Abbey CK, Borowsky AD, Gregg JP, Cardiff RD, Cherry SR (2006). Preclinical imaging of mammary intraepithelial neoplasia with positron emission tomography. J Mammary Gland Biol Neoplasia.

[34] Brown RS, Leung JY, Fisher SJ, Frey KA, Ethier SP, Wahl RL (1996). Intratumoral distribution of tritiated-FDG in breast carcinoma: correlation between Glut-1 expression and FDG uptake. J Nucl Med.

[35] Lin EY, Nguyen AV, Russell RG, Pollard JW (2001). Colony-stimulating factor 1 promotes progression of mammary tumors to malignancy. J Exp Med.

[36] Lin EY, Li JF, Gnatovskiy L, Deng Y, Zhu L, Grzesik DA (2006). Macrophages regulate the angiogenic switch in a mouse model of breast cancer. Cancer Res..

[37] DeNardo DG, Barreto JB, Andreu P, Vasquez L, Tawfik D, Kolhatkar N (2009). CD4(+) T cells regulate pulmonary metastasis of mammary carcinomas by enhancing protumor properties of macrophages. Cancer Cell.

[38] Fridlender ZG, Sun J, Kim S, Kapoor V, Cheng G, Ling L (2009). Polarization of tumor-associated neutrophil phenotype by TGF-beta: “N1” versus “N2” TAN. Cancer Cell.

[39] Mishalian I, Bayuh R, Levy L, Zolotarov L, Michaeli J, Fridlender ZG (2013). Tumor-associated neutrophils (TAN) develop pro-tumorigenic properties during tumor progression. Cancer Immunol Immunother.

[40] Shishido S, Delahaye A, Beck A, Nguyen TA (2013). The MMTV-PyVT transgenic mouse as a multistage model for mammary carcinoma and the efficacy of antineoplastic treatment. J Cancer Ther..

[41] Qian B-ZZ, Pollard JW (2010). Macrophage diversity enhances tumor progression and metastasis. Cell.

[42] Nozawa H, Chiu C, Hanahan D (2006). Infiltrating neutrophils mediate the initial angiogenic switch in a mouse model of multistage carcinogenesis. Proc Natl Acad Sci.

[43] Fridlender ZG, Albelda SM (2012). Tumor-associated neutrophils: friend or foe?. Carcinogenesis.

[44] Piccard H, Muschel RJ, Opdenakker G (2012). On the dual roles and polarized phenotypes of neutrophils in tumor development and progression. Crit Rev Oncol Hematol.

[45] Powell DR, Huttenlocher A (2016). Neutrophils in the tumor microenvironment. Trends Immunol.

[46] Grinnell F (1982). Migration of human neutrophils in hydrated collagen lattices. J Cell Sci..

[47] Monboisse JC, Garnotel R, Randoux A (1991). Adhesion of human neutrophils to and activation by type-I collagen involving a beta 2 integrin. J Leukoc Biol..

[48] Weathington NM, van Houwelingen AH (2006). A novel peptide CXCR ligand derived from extracellular matrix degradation during airway inflammation. Nat Med..

[49] Werr J, Johansson J, Eriksson EE, Hedqvist P (2000). Integrin α2β1 (VLA-2) is a principal receptor used by neutrophils for locomotion in extravascular tissue. Blood.

[50] Ridger VC, Wagner BE, Wallace WAH (2001). Differential effects of CD18, CD29, and CD49 integrin subunit inhibition on neutrophil migration in pulmonary inflammation. J Immunol..

[51] Lämmermann T, Bader BL, Monkley SJ, Worbs T, Wedlich-Söldner R, Hirsch K (2008). Rapid leukocyte migration by integrin-independent flowing and squeezing. Nature.

[52] Afonso PV, McCann CP, Kapnick SM, Parent CA (2013). Discoidin domain receptor 2 regulates neutrophil chemotaxis in 3D collagen matrices. Blood.

[53] Gomez-Cambronero J, Horn J, Paul CC (2003). Granulocyte-macrophage colony-stimulating factor is a chemoattractant cytokine for human neutrophils: involvement of the ribosomal p70 S6 kinase signaling pathway. J Immunol..

[54] Khajah M, Millen B, Cara DC (2011). Granulocyte-macrophage colony-stimulating factor (GM-CSF): a chemoattractive agent for murine leukocytes in vivo. J Leukoc Biol..

[55] Ferlazzo G, Klein J, Paliard X, Wei WZ (2000). Dendritic cells generated from CD34+ progenitor cells with flt3 ligand, c-kit ligand, GM-CSF, IL-4, and TNF-α are functional antigen-presenting cells resembling mature monocyte-derived dendritic cells. J Immunother.

[56] Dranoff G, Jaffee E, Lazenby A (1993). Vaccination with irradiated tumor cells engineered to secrete murine granulocyte-macrophage colony-stimulating factor stimulates potent, specific, and long-lasting anti-tumor immunity. Proc Natl Acad Sci..

[57] Eubank TD, Roberts RD, Khan M, Curry JM, Nuovo GJ, Kuppusamy P (2009). Granulocyte macrophage colony-stimulating factor inhibits breast cancer growth and metastasis by invoking an anti-angiogenic program in tumor-educated macrophages. Cancer Res.

[58] Morales JK, Kmieciak M, Knutson KL, Bear HD, Manjili MH (2010). GM-CSF is one of the main breast tumor-derived soluble factors involved in the differentiation of CD11b-Gr1- bone marrow progenitor cells into myeloid-derived suppressor cells. Breast Cancer Res Treat..

[59] Ghirelli C, Reyal F, Jeanmougin M, Zollinger R, Sirven P, Michea P (2015). Breast cancer cell-derived GM-CSF licenses regulatory Th2 induction by plasmacytoid predendritic cells in aggressive disease subtypes. Cancer Res.

[60] Heldin CH (2013). Targeting the PDGF signaling pathway in tumor treatment. Cell Commun Signal..

[61] Shao ZM, Nguyen M, Barsky SH (2000). Human breast carcinoma desmoplasia is PDGF initiated. Oncogene.

[62] Deuel TF, Senior RM, Huang JS, Griffin GL (1982). Chemotaxis of monocytes and neutrophils to platelet-derived growth factor. J Clin Invest..

[63] Tzeng DY, Deuel TF, Huang JS, Senior RM, Boxer LA, Baehner RL (1984). Platelet-derived growth factor promotes polymorphonuclear leukocyte activation. Blood.

[64] Nozaki S, Sledge GW, Nakshatri H (2000). Cancer cell-derived interleukin 1alpha contributes to autocrine and paracrine induction of pro-metastatic genes in breast cancer. Biochem Biophys Res Commun.

[65] Ji H, Houghton AM, Mariani TJ, Perera S, Kim CB, Padera R (2006). K-ras activation generates an inflammatory response in lung tumors. Oncogene.

[66] Sparmann A, Bar-Sagi D (2004). Ras-induced interleukin-8 expression plays a critical role in tumor growth and angiogenesis. Cancer Cell.

[67] Luboshits G, Shina S, Kaplan O, Engelberg S, Nass D, Lifshitz-Mercer B (1999). Elevated expression of the CC chemokine regulated on activation, normal T cell expressed and secreted (RANTES) in advanced breast carcinoma. Cancer Res.

[68] Adler EP, Lemken CA, Katchen NS, Kurt RA (2003). A dual role for tumor-derived chemokine RANTES (CCL5). Immunol Lett.

[69] Stormes KA, Lemken CA, Lepre JV, Marinucci MN, Kurt RA (2005). Inhibition of metastasis by inhibition of tumor-derived CCL5. Breast Cancer Res Treat.

[70] Zhang Y, Lv D, Kim H-JJ, Kurt RA, Bu W, Li Y (2013). A novel role of hematopoietic CCL5 in promoting triple-negative mammary tumor progression by regulating generation of myeloid-derived suppressor cells. Cell Res.

[71] Venmar KT, Fingleton B (2014). Lessons from immunology: IL4R directly promotes mammary tumor metastasis. Oncoimmunology.

[72] Venmar KT, Carter KJ, Hwang DG, Dozier EA, Fingleton B (2014). IL4 receptor ILR4α regulates metastatic colonization by mammary tumors through multiple signaling pathways. Cancer Res.

[73] Taub DD, Conlon K, Lloyd AR, Oppenheim JJ (1993). Preferential migration of activated CD4+ and CD8+ T cells in response to MIP-1 alpha and MIP-1 beta. Science..

[74] Nath A, Chattopadhya S, Chattopadhyay U, Sharma NK (2006). Macrophage inflammatory protein (MIP)1alpha and MIP1beta differentially regulate release of inflammatory cytokines and generation of tumoricidal monocytes in malignancy. Cancer Immunol Immunother.

[75] Kitamura T, Qian B-ZZ, Soong D, Cassetta L, Noy R, Sugano G (2015). CCL2-induced chemokine cascade promotes breast cancer metastasis by enhancing retention of metastasis-associated macrophages. J Exp Med.

[76] Shankaran V, Ikeda H, Bruce AT, White JM, Swanson PE, Old LJ (2001). IFNgamma and lymphocytes prevent primary tumour development and shape tumour immunogenicity. Nature.

