# Supplementary figures and images for: Neutrophils drive accelerated tumor progression in the collagen-dense mammary tumor microenvironment
Source: Breast Cancer Res. 2016 May 11;18:49. doi: 10.1186/s13058-016-0703-7 (PMC4864897; doi:10.1186/s13058-016-0703-7)

# TUMOR

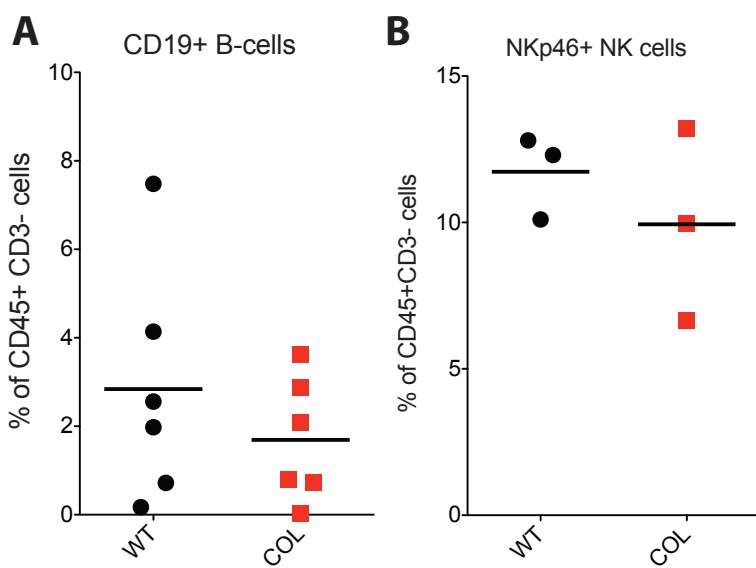

# SPLEEN

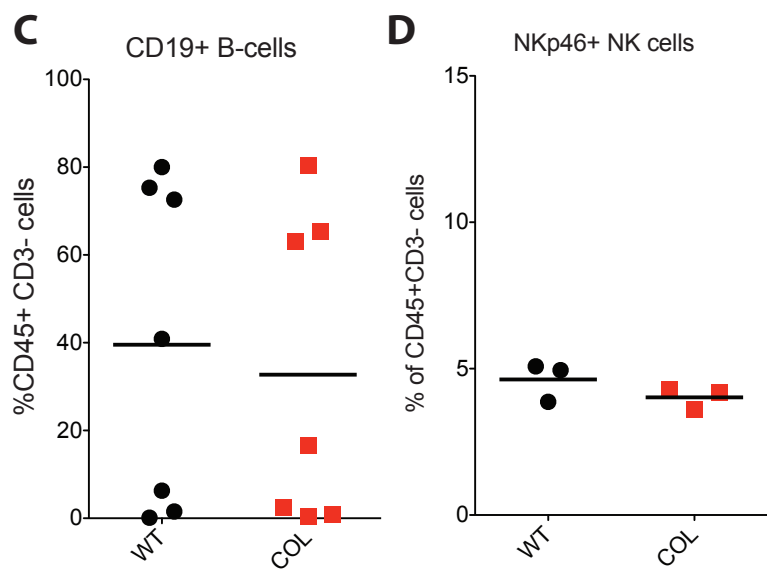

Supplement: Additional file 1: — Quantification of B cell and NK cells found in late-stage mammary tumors. A The percentage of CD45+CD3- cells that are CD19+ B cells in WT and COL tumors and C spleens at 15 weeks. B The percentage of CD45+CD3- cells that are Nkp46+ NK cells in WT and COL tumors and D spleens at 15 weeks. (PDF 293 kb) [file 13058_2016_703_MOESM1_ESM.pdf]

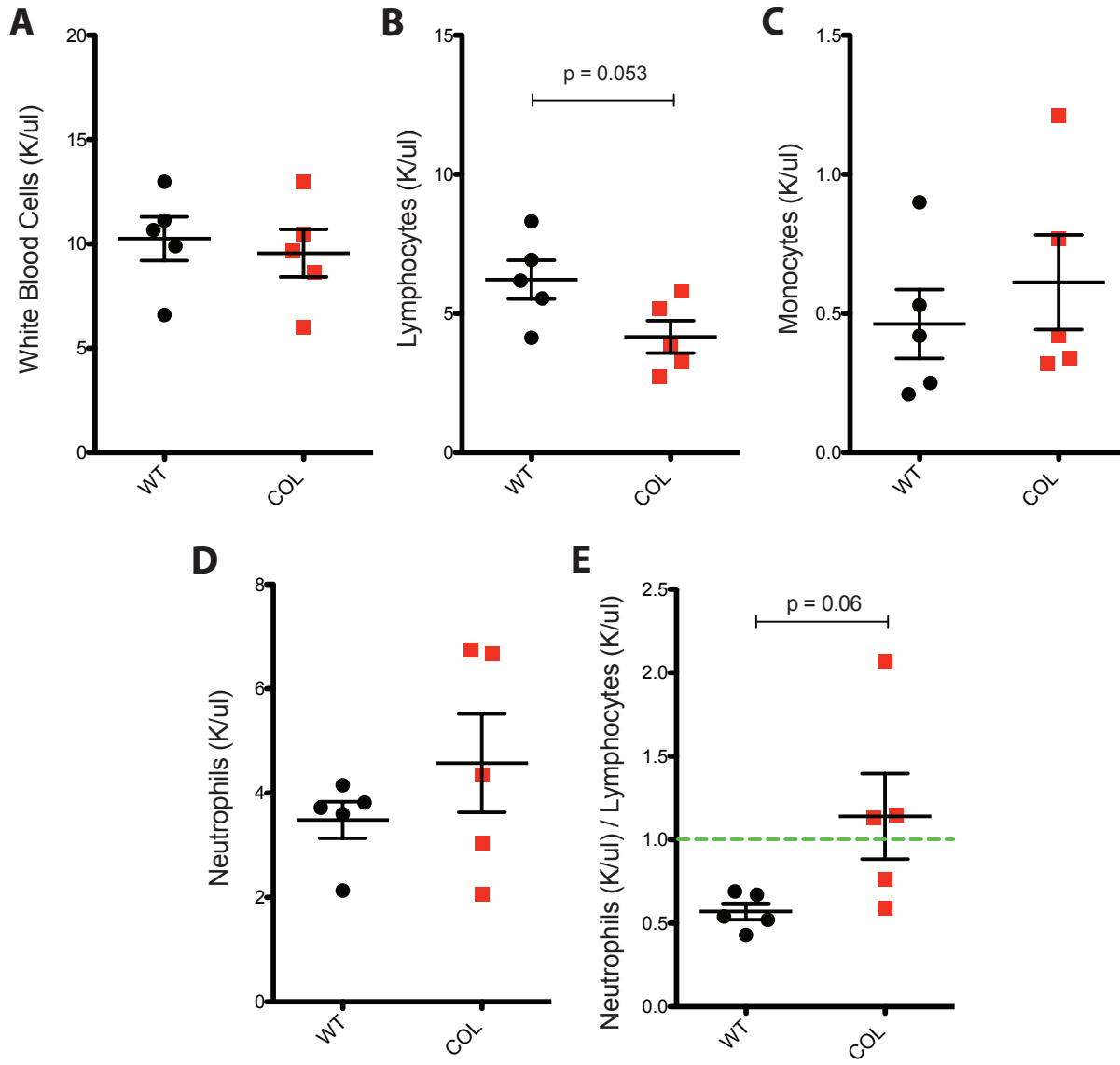

Supplement: Additional file 2: — Tumor mice complete blood counts at late tumor stages. A White blood cell, B lymphocyte, C monocyte, and D neutrophil counts in 103 per μl of blood from WT and COL tumor mice at 15 weeks. Blood was obtained by a retro-orbital bleeding procedure and counted on a Hemavet 950FS hematology system. E Neutrophil to lymphocyte ratio (NLR) a.u. Green dash line denotes no change (NLR = 1) (n = 5). (PDF 311 kb) [file 13058_2016_703_MOESM2_ESM.pdf]

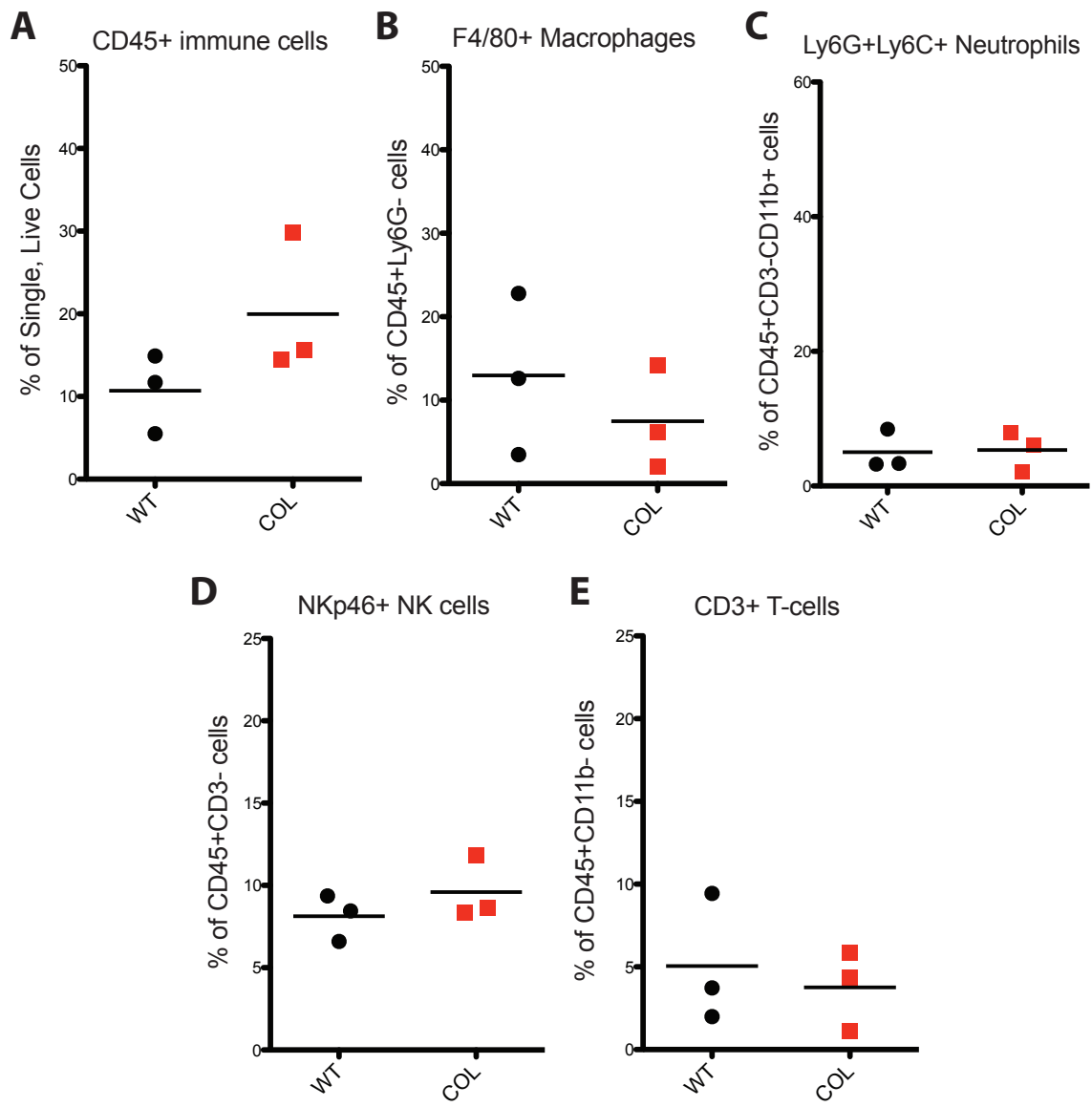

Supplement: Additional file 3: — Quantification of immune cells in wild-type and collagen-dense non-tumor mammary glands. A Percentage of CD45+ immune cells in WT and COL mammary glands at 15 weeks (n = 3, of which each is an independent pair of age-matched littermate WT and COL non-tumor mice). B Percentage of CD45+CD3-CD11b+ cells that are F4/80+ macrophages in mammary glands. C Percentage of CD45+CD3-CD11b+ cells that are Ly6G+Ly6C+ neutrophils. D Percentage of CD45+CD3- cells that are Nkp46+ NK cells. E Percentage of CD45+CD3+ T-cells in mammary glands, determined by flow cytometry as in Fig. 1. (PDF 313 kb) [file 13058_2016_703_MOESM3_ESM.pdf]

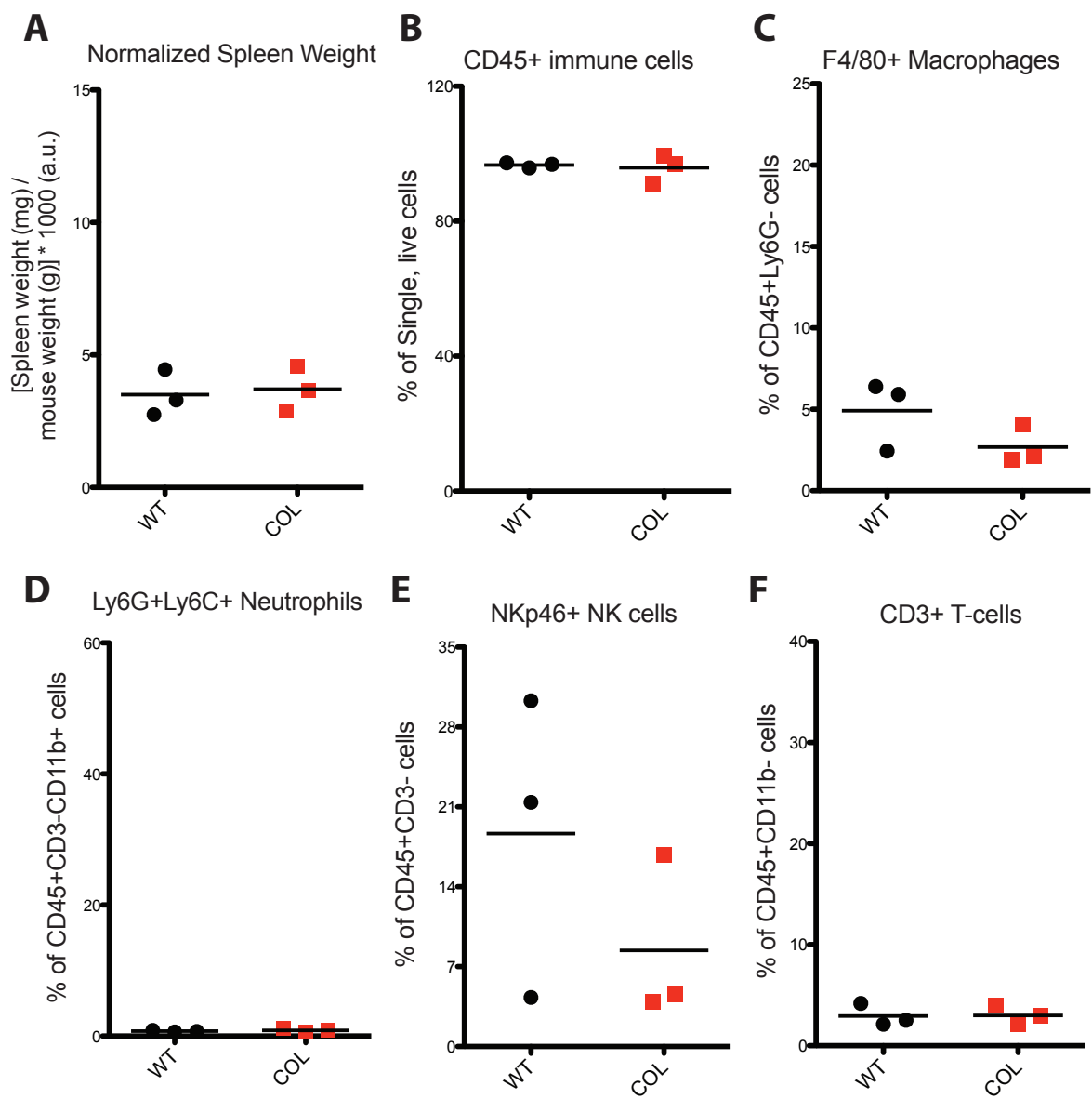

Supplement: Additional file 4: — Quantification of immune cells in spleens from wild-type and collagen-dense non-tumor mice. A Spleen weight (mg) normalized to mouse weight (g) from both WT and COL mice (n = 3). B Percentage of CD45+ immune cells, C CD45+CD3-CD11b+ cells that are F4/80+, D CD45+CD3-CD11b+ cells that are Ly6G+Ly6C+ neutrophils, E CD45+CD3-CD11b+ cells that are Nkp46+ NK cells, and F CD45+CD3+ T cells found in WT and COL spleens from non-tumor mice at 15 weeks, determined by flow cytometry as in Additional file 3. (PDF 327 kb) [file 13058_2016_703_MOESM4_ESM.pdf]

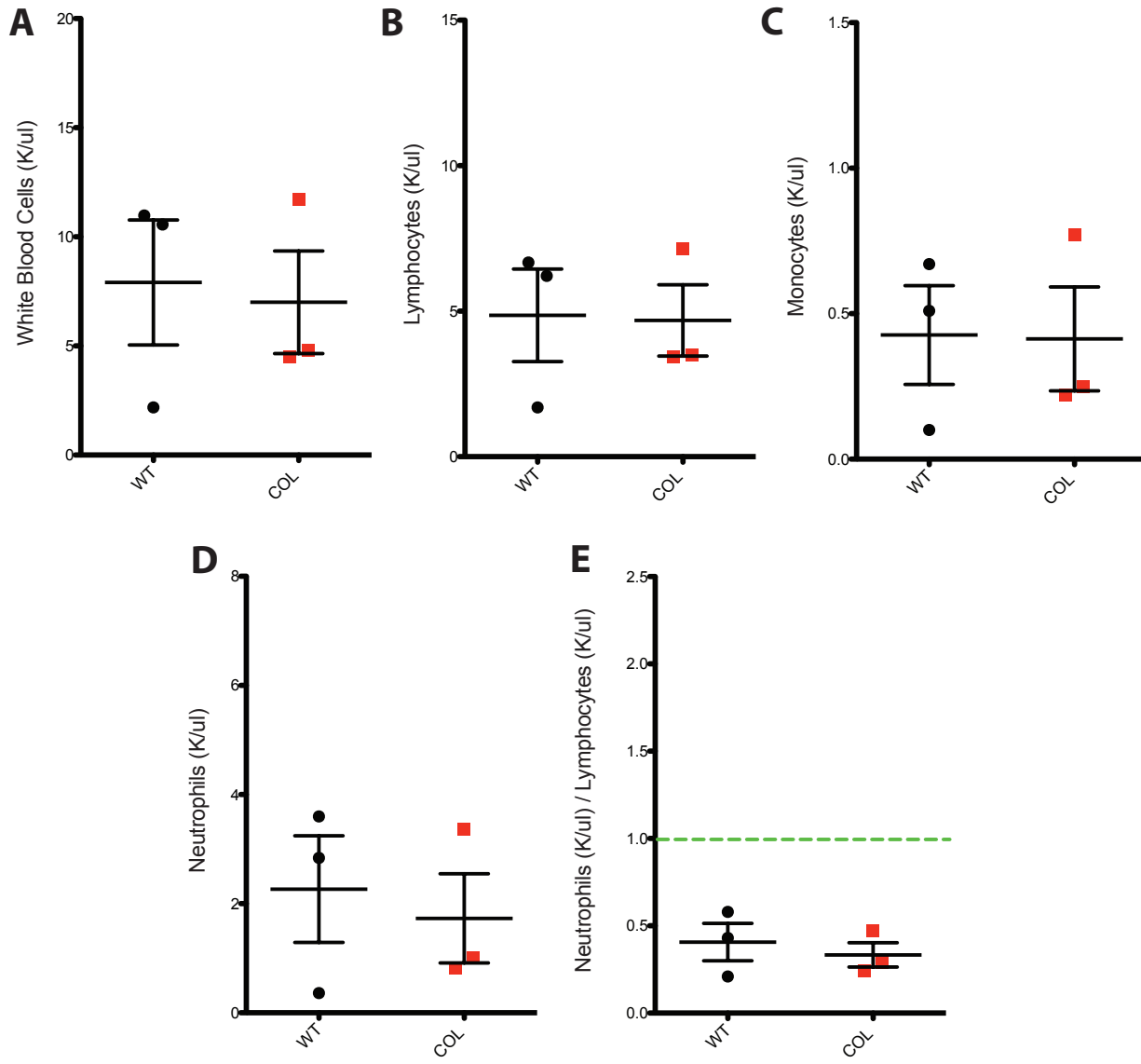

Supplement: Additional file 5: — Non-tumor mice complete blood counts at 15 weeks of age. A White blood cell, B lymphocyte, C monocyte, and D neutrophil counts in 103 per μl of blood from WT and COL non-tumor. Blood was obtained as in Additional file 2. E Neutrophil to lymphocyte ratio (NLR) a.u. Green dash line denotes no change (NLR = 1) (n = 3). (PDF 304 kb) [file 13058_2016_703_MOESM5_ESM.pdf]

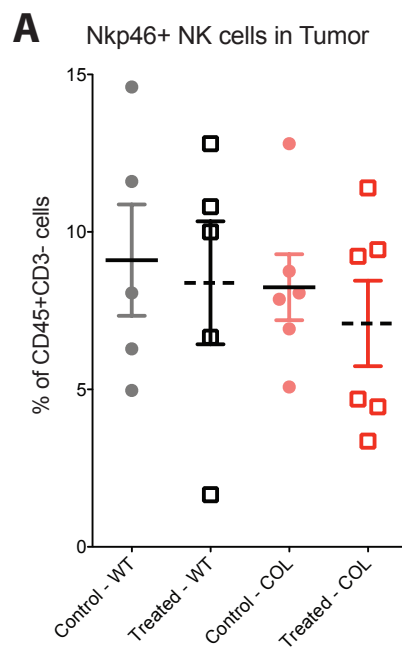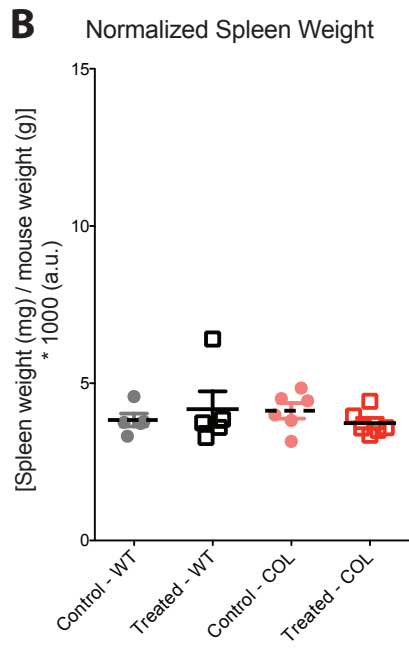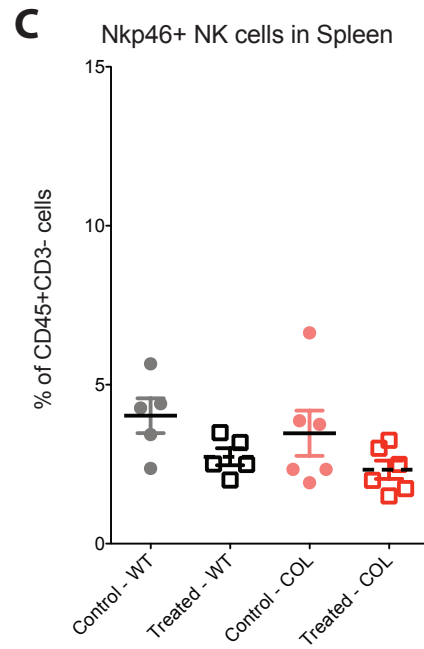

Supplement: Additional file 6: — Recruitment NK cells in neutrophil-depleted tissues. A The percentage of CD45+CD3– cells that are Nkp46+ NK cells in anti-Ly6G treated or IgG control WT and COL tumors at 12 weeks. B Spleen weight (mg) normalized to mouse weight (g) from anti-Ly6G treated and IgG control WT and COL tumor mice. C The percentage of CD45+CD3– cells that are Nkp46+ NK cells in anti-Ly6G treated or IgG control WT and COL spleens at 12 weeks. (PDF 300 kb) [file 13058_2016_703_MOESM6_ESM.pdf]

**A**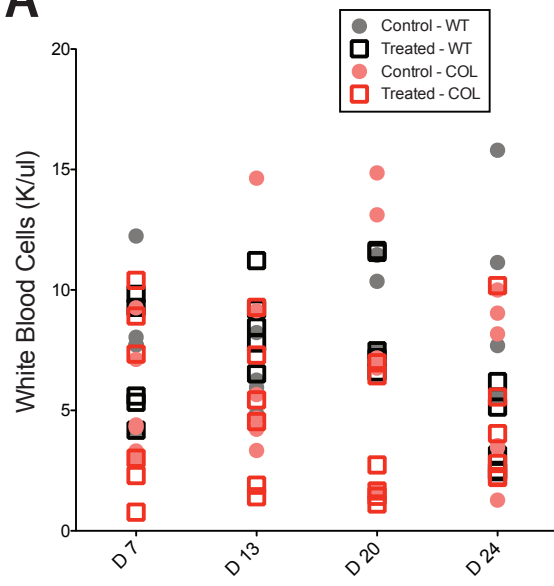**B**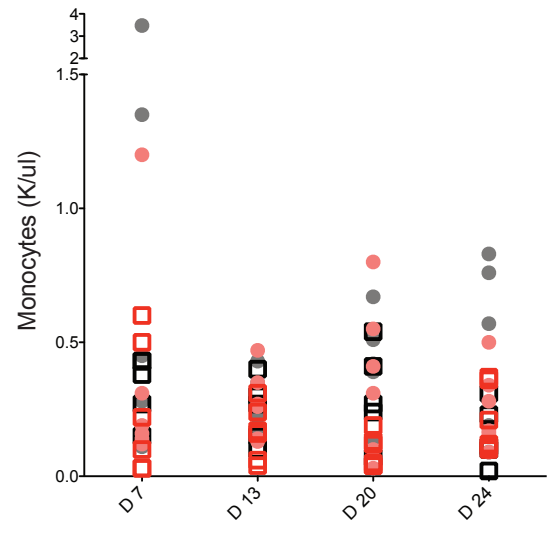**C**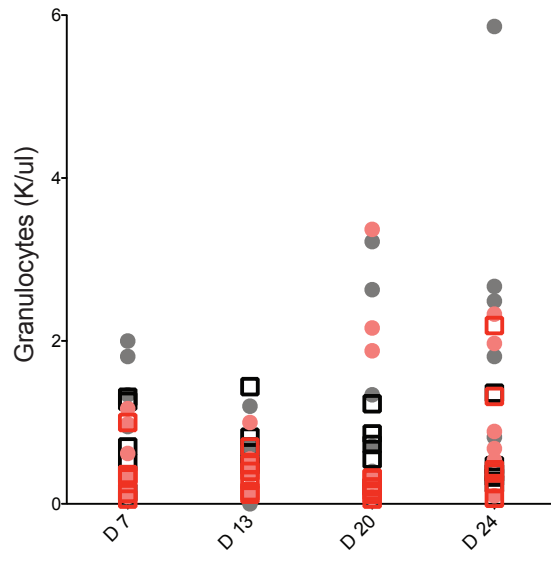**D**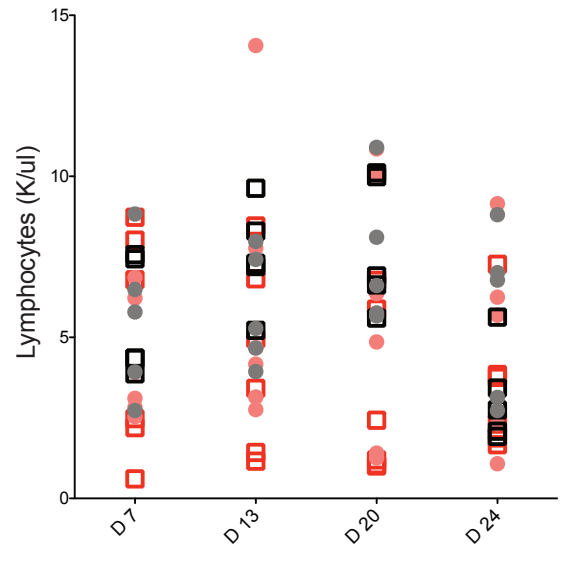**E**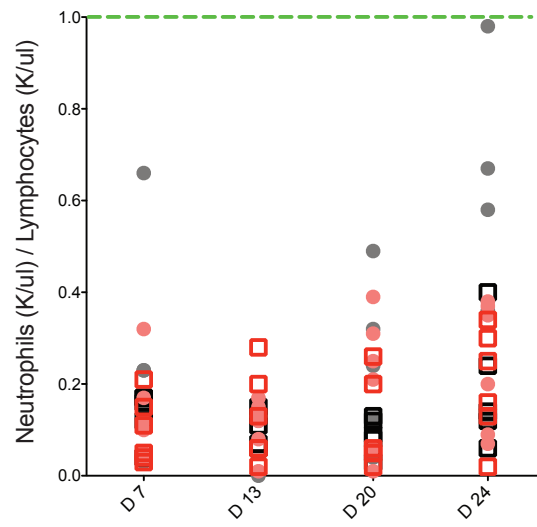

Supplement: Additional file 7: — Complete blood counts at different time points during neutrophil depleting study. A White blood cell, B monocyte, C granulocyte, and D lymphocyte count in 103 per μl from treated and control WT and COL mice obtained by saphenous vein bleeding, and counted on a Hemavet 950FS hematology system. E Neutrophil to lymphocyte ratio (NLR) a.u. (n =5 or 6). (PDF 336 kb) [file 13058_2016_703_MOESM7_ESM.pdf]

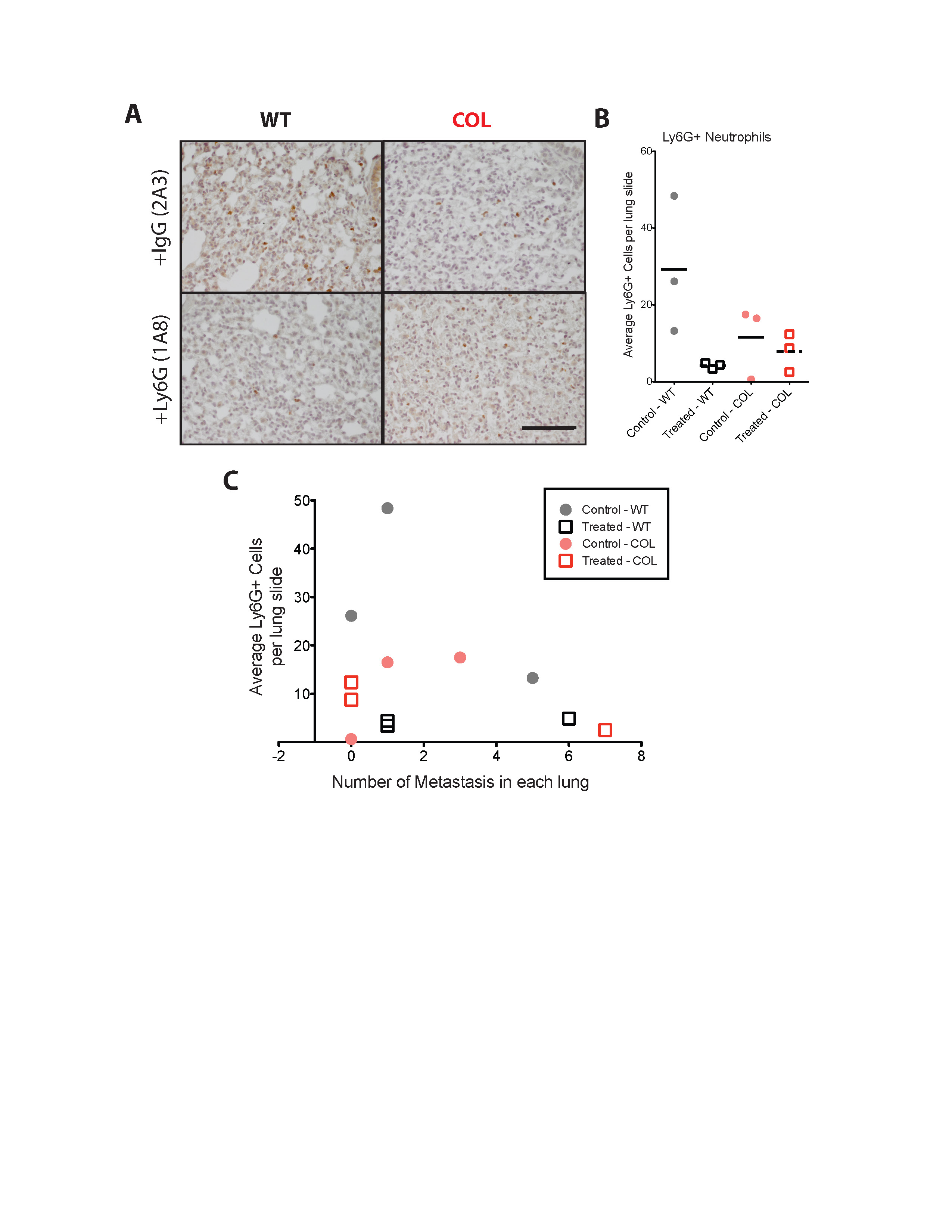

Supplement: Additional file 8: — Neutrophil counts in metastatic lungs do not correlate with number of metastases in depletion study. A Immunohistochemical analysis of lungs from the four treatment groups. Lungs were stained with Ly6G (1A8) in DAB and counterstained with hematoxylin. Scare bar 100 um. B Average number of Ly6G+ cells found in eight fields of view per slide (n = 3). C Average number of Ly6G+ cells per lung versus the number of metastatic lesions per lungs. (JPG 558 kb) [file 13058_2016_703_MOESM8_ESM.jpg]
